# Glutathione-S-Transferases in the Olfactory Organ of the Noctuid Moth *Spodoptera littoralis*, Diversity and Conservation of Chemosensory Clades

**DOI:** 10.3389/fphys.2018.01283

**Published:** 2018-09-27

**Authors:** Nicolas Durand, Marie-Anne Pottier, David Siaussat, Françoise Bozzolan, Martine Maïbèche, Thomas Chertemps

**Affiliations:** Sorbonne Université, INRA, CNRS, UPEC, IRD, Univ. P7, Institute of Ecology and Environmental Sciences of Paris, Paris, France

**Keywords:** GST (glutathione S transferase), *Spodoptera littoralis*, olfaction, detoxification, odorant degrading enzyme

## Abstract

Glutathione-S-transferases (GSTs) are conjugating enzymes involved in the detoxification of a wide range of xenobiotic compounds. The expression of GSTs as well as their activities have been also highlighted in the olfactory organs of several species, including insects, where they could play a role in the signal termination and in odorant clearance. Using a transcriptomic approach, we identified 33 putative GSTs expressed in the antennae of the cotton leafworm *Spodoptera littoralis*. We established their expression patterns and revealed four olfactory-enriched genes in adults. In order to investigate the evolution of antennal GST repertoires in moths, we re-annotated antennal transcripts corresponding to GSTs in two moth and one coleopteran species. We performed a large phylogenetic analysis that revealed an unsuspected structural—and potentially functional—diversity of GSTs within the olfactory organ of insects. This led us to identify a conserved clade containing most of the already identified antennal-specific and antennal-enriched GSTs from moths. In addition, for all the sequences from this clade, we were able to identify a signal peptide, which is an unusual structural feature for GSTs. Taken together, these data highlight the diversity and evolution of GSTs in the olfactory organ of a pest species and more generally in the olfactory system of moths, and also the conservation of putative extracellular members across multiple insect orders.

## Introduction

Detoxification is a common process present in nearly all living organisms, from prokaryotes to eukaryotes, allowing the elimination of toxic substances of both exogenous or endogenous origin by sequential enzymatic reactions. The first step of detoxification consists in the introduction of functional groups into lipophilic xenobiotics, mainly by oxido-reduction and hydrolysis reactions performed by phase I enzymes, such as cytochromes P450s (CYPs) and carboxylesterases (CCEs). These phase I metabolites are then conjugated to small hydrophilic molecules by phase II enzymes, a large group of broad-specificity transferases, which in combination can metabolize almost any hydrophobic compound that contains nucleophilic or electrophilic groups (Bock, 2010). Two of the most important classes of this group are the Glutathione-S-transferases (GSTs, EC 2.5.1.18), and the Uridine diphosphate-glycosyltransferases (UGTs, EC 2.4.1.17) (Jakoby and Ziegler, 1990). These two steps of biotransformation led to more hydrophilic metabolites, facilitating their final excretion by various efflux transporters (phase III) such as multidrug resistance transporters (Dermauw and Van Leeuwen, 2014).

Among phase II enzymes, GSTs are highly diversified and play various functions in the detoxification of a wide range of xenobiotic compounds but also in normal cellular metabolism or in oxidative stress response (Li et al., 2007). They catalyze the conjugation of tri-peptide glutathione (GSH) to endogenous electrophilic molecules or to products from the phase I. GSTs are hetero- or homo-dimeric proteins of approximately 25 kDa in size. Each monomer has two domains joined by a variable linker region. The amino terminal domain is highly conserved and provides most of the GSH binding site (G-site) while the carboxyl terminal domain interacts with the hydrophobic substrate (H-site).

In insects, GSTs can be sorted based on their cellular localizations, i.e., mainly cytosolic or microsomal, on their substrate specificities and phylogenetic relationships (Enayati et al., 2005). The cytosolic GSTs are divided in six classes (Delta, Epsilon, Omega, Sigma, Theta, and Zeta) (Sheehan et al., 2001). Despite that they catalyze similar reactions than cytosolic GSTs, microsomal GSTs, now referred as membrane-associated proteins in eicosanoid and glutathione (MAPEG), are very different in origin and structure, as they are mainly trimeric transmembrane proteins (Toba and Aigaki, 2000). Insect GSTs are intensely studied for their role in insecticide resistance (Enayati et al., 2005), in particular, members of the delta and epsilon classes, which are specific to arthropods, have been implicated in resistance to various pesticides. MAPEGs also play a role in xenobiotic detoxification (Zhou et al., 2013) and seem in addition involved in aging process (Toba and Aigaki, 2000). Omega, theta, zeta and microsomal sub-groups appear to be involved in various cellular processes, including protection against oxidative stress (Tu and Akgül, 2005).

Insect GSTs are known to be specifically or preferentially expressed into major detoxification organs such as fat body, midgut but also in epidermis and in Malpighian tubules (Huang et al., 2011). However, the expression of GSTs as well as their activities have been also highlighted in the olfactory organs of several insect species. Antennal expressed GSTs have been indeed identified in various moth species, such as *Manduca sexta* (Rogers et al., 1999), *Helicoverpa armigera* (Wang et al., 2004), *Amyelois transitella* (Leal et al., 2009), *Bombyx mori* (Tan et al., 2014), *Chilo suppressalis* (Liu et al., 2015a), *Epiphyas postvittana* (Corcoran et al., 2015), *Cnaphalocris medinalis* (Liu et al., 2015b), and *Cydia pomonella* (Huang et al., 2017), but also in the fruit fly *Drosophila melanogaster* (Younus et al., 2014) or in the beetles *Agrilus planipennis* (Mamidala et al., 2013), *Dendroctonus valens* (Gu et al., 2015), and *Phyllotreta striolata* (Wu et al., 2016). This particular localization led to the hypothesis of a possible dual function of GSTs in antennae where, besides their original implication in xenobiotic metabolism, they could play a role in the signal termination and in odorant clearance, as Odorant-Degrading Enzymes (ODEs, Vogt and Riddiford, 1981; Chertemps, 2017). Moreover, the biochemical characterization of an antennal-restricted GST in *M. sexta* (*msexGST-msolf*) confirmed the activity of such enzymes in odorant conjugation and highlighted the possible contribution of GSTs to the detoxification of compounds that might interfere with odorant detection (Rogers et al., 1999).

The cotton leaf worm *Spodoptera littoralis*, is a highly polyphagous crop pest in a broad area of distribution around the Mediterranean basin (Salama et al., 1971). In this pest species, transcriptomic approaches (Legeai et al., 2011; Jacquin-Joly et al., 2012) have already led to the identification of various olfactory gene repertoires, such as Odorant Receptors and Odorant-Binding Proteins, but also of several phase I and II enzymes, such as CCEs (Durand et al., 2010b), CYPs (Pottier et al., 2012), or UGTs (Bozzolan et al., 2014). Here, using more recent and complete transcriptomic data (Poivet et al., 2013), we identified 33 putative GSTs expressed in the antennae of this species. Moreover, their expression patterns were studied using both qualitative and quantitative PCR and revealed four olfactory-enriched genes in *S. littoralis* adults. In order to investigate the evolution and phylogenetic relationships of antennal GST repertoires in moths and the relative conservation of such diversity, we built a phylogenetic analysis based on available sequences from 18 insect species, including 13 lepidopteran species. We first re-annotated antennal transcripts corresponding to GSTs in two other moth species belonging to basal lepidopteran taxa, i.e., the peach fruit moth, *Carposina sasakii* from the Carposinidae family and the purplish birch-miner moth *Eriocrania semipurpurella*, as a member of the Eriocraniidae. We then annotated GSTs from an antennal transcriptome of a coleopteran species, the european spruce bark beetle *Ips typographus*, to compare with non-lepidopteran species. This large phylogenetic analysis revealed an unsuspected structural—and potentially functional—diversity of GSTs within the olfactory organ. Surprisingly, inside the delta class, we identified a conserved clade containing most of the already identified antennal-specific and antennal-enriched GSTs from moths. In addition, for all the sequences from this clade, we were able to identify a signal peptide, which is an unusual structural feature for GSTs. Taken together, these data highlight the diversity and evolution of GSTs in the olfactory organ of a pest species and more generally in the olfactory system of moths, with in particular the finding of some conserved putative extracellular members across multiple insect orders.

## Materials and methods

### Insects and tissue collection

Insects were reared on semi-artificial diet at 23°C, 60–70% relative humidity, and under a 16:8 h light: dark (LD) photoperiod. Adults were kept under an inverted LD regime and provided with a 10% sucrose solution. Male and female antennae and various tissues (proboscis, brain, legs, thorax and abdomen) from 2 day-old males as well as from 7th instar larvae (heads, guts and carcasses) were dissected and stored at −80°C until RNA extraction.

### RNA isolation and cDNA synthesis

Total RNAs were extracted with TRIzol®Reagent (Invitrogen, Carlsbad, CA, United States) and were quantified by spectrophotometry at 260 nm. Single-stranded cDNAs were synthesized from total RNAs (5 μg) from the various tissues using Superscript II reverse transcriptase (Gibco BRL, Invitrogen) and an oligo(dT)18 primer and they were treated with DNase I (Roche, Basel, Switzerland).

### RT-PCR and qRT-PCR

Tissue distribution of *S. littoralis* GSTs was first investigated by RT-PCR. The ubiquitous ribosomal *SlitRpl13* gene, which presents a constant expression in all tissues tested (Durand et al., 2010a), was used as control gene. Primer pairs and PCR conditions are indicated in Table S1. PCR products were loaded on 1% agarose gels and visualized using Gel Red (VWR, Radnor, PE, United States) (Figure S2). Amplification by qPCR of 8 *S. littoralis* GSTs, namely *SlitGSTd2, SlitGSTe6, SlitGSTe8, SlitGSTe9, SlitGSTe15, SlitGSTs6, SlitGSTo3, SlitMGST1-3*, and the reference gene *SlitRpl13* was performed as described in detail in Durand et al. (2010a) using the LightCycler® 480 real-time PCR system (Roche). Data were analyzed with LightCycler 480® software (Roche). The crossing point values (C_p_-values) were first determined for the reference genes with a run formed by the 5-fold dilution series, the measuring points, and three negative controls. The normalized *S. littoralis* GST expressions were calculated with Q-Gene software (Simon, 2003) using *SlitRpl13* as reference. This gene has been already demonstrated as the best reference gene in these conditions (Durand et al., 2010b). Each reaction was run in triplicate (technical replicate) with three independent biological replicates. Statistical analyses have been performed with GraphPad Prism®5 software (ANOVA, post-test Tukey's multiple comparison).

### Identification of antennal GSTs

Putative partial GST cDNAs were identified from a *de novo* transcriptome of *S. littoralis* (Poivet et al., 2013) by tBLASTn against a dataset of 47 *Spodoptera litura* GST sequences (Zhang et al., 2016) and using known GST genes from insect non-redundant sequence databases (National Center for Biotechnology Information, NCBI). Sequences were completed with the *de novo* transcriptome of *S. littoralis* composed of tissues from various origin, including larvae (Poivet et al., 2013). Antennal enriched sequences where then deduced from antennal specific transcriptomes (Legeai et al., 2011), and confirmed with PCR methods (see above). We named all *S. littoralis* sequences according to the corresponding *S. litura* and *S. frugiperda* sequences (Zhang et al., 2016; Gouin et al., 2017). Subsequently the 7 *S. littoralis* GSTs that were previously published in (Lalouette et al., 2016), namely *SlGSTe1, e2, e3, e4, d1, d2*, and *d3* were respectively renamed *SlitGSTe15, e8, e14, e12, d2, d3*, and *SlitMGST1-3*. All sequences have been deposited in Genbank with reference accession number from MH177577 to 177613. GST sequences from other antennal transcriptomes were retrieve from high quality datasets (regarding length and quality of transcripts and/or number of overall sequences) and selected in various clades of holometabolous insects for comparison. *Ips typographus, E. semipurpurella*, and *C. sasakii* were manually annotated from published antennal transcriptomes [PRJEB3262, PRJNA377940, (Yuvaraj et al., 2017), and PRJNA383289 respectively] using the same protocol as *S. littoralis* sequences and named according our phylogenetic analysis (Figure S3).

### Multiple sequence alignments and phylogenetic analysis

Amino acid sequences were aligned using MAFFT (using L-INS-i option) (Katoh and Standley, 2013) implemented in the Geneious software (http://www.geneious.com, Kearse et al., 2012). Phylogenetic trees were constructed using PhyML (Guindon et al., 2010) based on the LG+G+I+F substitution model as determined by the SMS server (Lefort et al., 2017), using Nearest Neighbor Interchange (NNI). Branch supports were estimated by a Bayesian-like transformation of aLRT (aBayes) (Anisimova et al., 2011). A dendrogram was created and colored using FigTree software (http://tree.bio.ed.ac.uk/software/figtree/). Our final dataset included 415 sequences, including 271 sequences from 13 lepidopteran species for the cytosolic GST sequences, and 15 sequences from 6 lepidopteran species for the MAPEGs.

### Identification of predicted signal peptides in GST sequences

SignalP4.1 software (Petersen et al., 2011) was used with default D-cutoff value to predict the presence and location of signal peptide (SP) cleavage sites in the GST amino acid sequences. We performed a tBlastn analysis on the nucleotide collection and transcriptome shotgun assembly databases available on the NCBI website. To provide a clear representation of the diversity of GSTs with SP, we aligned a selection of sequences corresponding to the different organism families using MUSCLE (Edgar, 2004) (Figure S4).

## Results

### Identification of *S. littoralis* antennal GSTs

**Figure 1:**
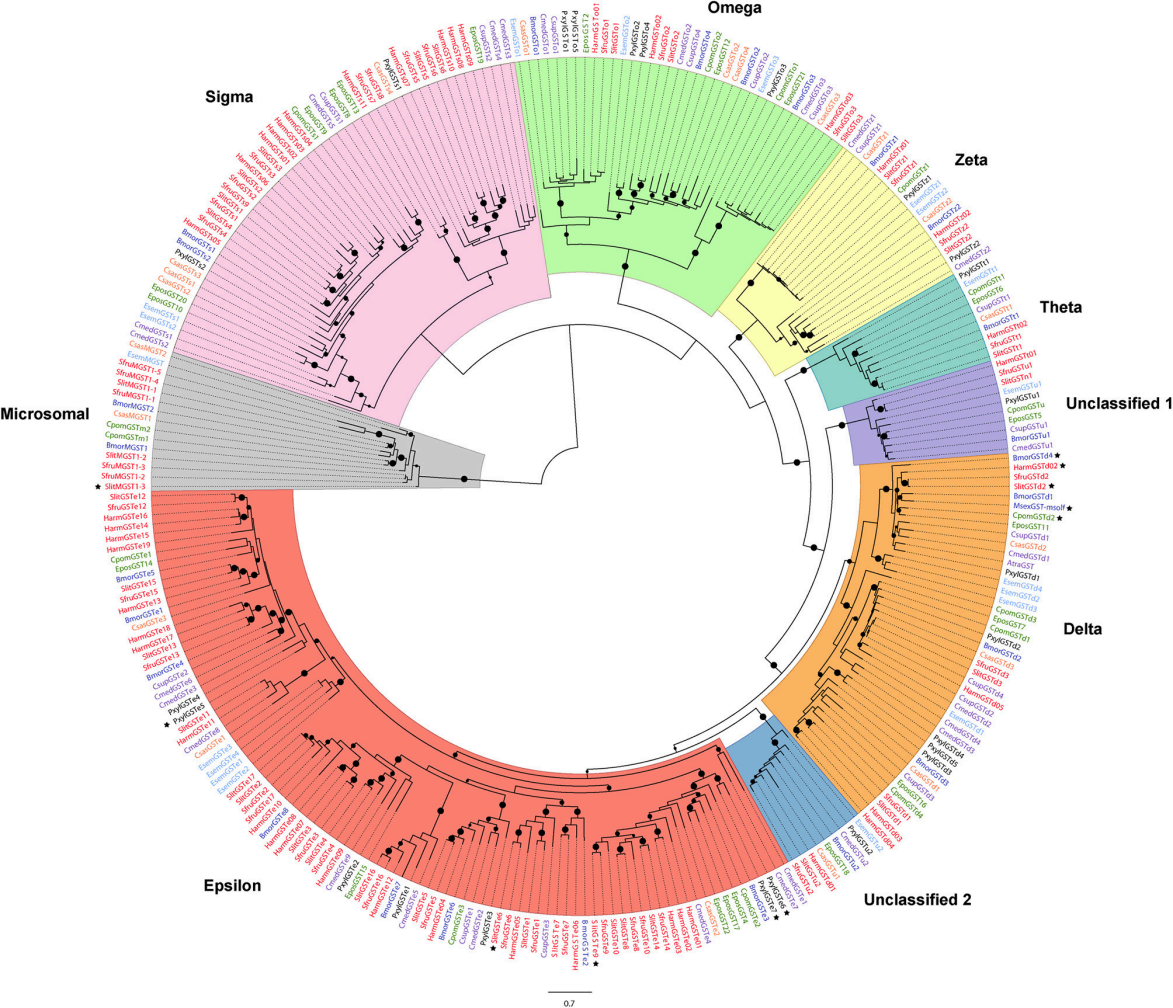
Maximum-likelihood phylogeny of antennal-expressed lepidopteran GSTs. The tree was built from amino-acid sequences of GST repertoires of *S. littoralis, S. frugiperda, H. armigera* (Noctuidae family, branches colored in red), *M. sexta, B. mori* (Bombycoidea, dark blue), *C. pomonella, E. postvittana* (Tortricoidea; green), *C. medinalis*, C. *suppressalis, A. transitella* (Pyraloidea, purple), *C. sasakii* (Carposinidae, orange), *P. xylostella* (Plutellidae, black), and *E. semipurpurella* (Eriocraniidae, light blue). The MAPEG clade was used as an outgroup. Circles represent nodes highly supported by the likelihood-ratio test (small dots: aLRT > 0.9, middle dots: aLRT > 0.95, big dots: aLRT = 1). The black stars indicate antennal-enriched or antennal-specific GSTs. The scale bar represents 0.7 expected amino-acid substitutions per site.

A total of 37 full-length sequences encoding putative GST proteins from *S. littoralis* (SlitGSTs) were identified in our transcriptomic analysis, including 30 cytosolic GSTs and 3 MAPEGs expressed in antennae (Table 1 and Figure 1). Their molecular characteristics such as peptide lengths, estimated molecular masses as well as isoelectric points are indicated in Table S2. As a comparison, we analyzed several available transcriptomes from various holometabolous insects. As shown by the comparison with antennal sequences from other species listed in Table 1, this is the highest number of putative GSTs identified in an insect antenna. An overlook of GSTs in other holometabolous insects, such as Diptera and Coleoptera, confirmed this high number with only 19 sequences in *P. striolata* (Wu et al., 2016), 17 in *I. typographus* (this study) and 31 in *D. melanogaster* (Younus et al., 2014). The high number of antennal GSTs in *S. littoralis* is mainly due to an expansion of the epsilon clade, with 15 GSTe sequences for only one to six in the other moth species (Table 1), a phenomenon also observed in *D. melanogaster* with 12 GSTe (Younus et al., 2014).

**Table 1:** Comparison of antennal GSTs repertoire in insect species.

	**Family**	**Species**	**Delta**	**Epsilon**	**Zeta**	**Theta**	**Sigma**	**Omega**	**Unclassified**	**Microsomal**	**Total**	**Reference**
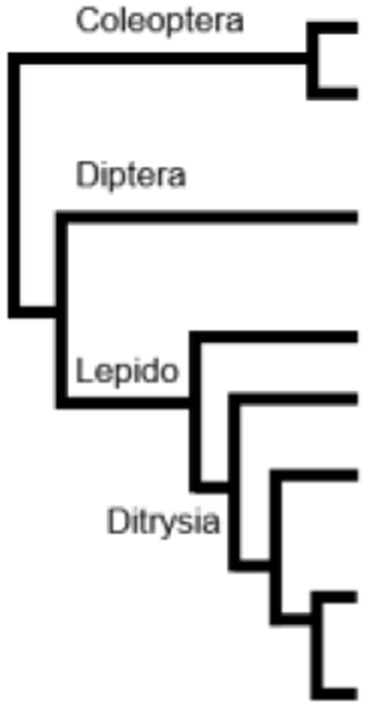	Chrysomelidae	*Phyllotreta striolata*	3(1)	6	1	1	4	1	1	2(2)	19	Wu et al., 2016^*^
	Curculionidae	*Ips typographus*	1	5	-	1	5	2	1	2	17	This study
	Drosophilidae	*Drosophila melanogaster*	8(2)	12(2)	2	4	1	3	1	-	31	Younus et al., 2014
	Eriocraniidae	*Eriocrania semipurpurella*	4	4	2	1	2	3	2	1	19	This study
	Carposinidae	*Carposina sasakii*	3	3	2	1	4	4	1	2	20	This study
	Tortricidae	*Cydia pomonella*	1(1)	1	1	1	1	2	1	2	10	Huang et al., 2017
		*Epiphyas postvittana*	3	5	-	1	6	3	2	-	20	Corcoran et al., 2015
	Crambidae	*Chilo suppressalis*	4	3	1	1	2	4	1	-	16	Liu et al., 2015a
		*Cnaphalocrocis medinalis*	4	5	2	-	5	3	2	-	21	Liu et al., 2015b
	Noctuidae	*Spodoptera littoralis*	3(1)	15(2)	2	1	4	3	2	3(1)	33	This study

*Numbers of adult antennal enriched GSTs are indicated between parentheses*.

* *Corrected number. Original number was 27 but 9 sequences are not insect GSTs and were removed from the analysis (Table S5)*.

### Phylogeny of lepidopteran antennal GSTs

As shown by phylogenetic analysis (Figure 1), all the identified sequences from *S. littoralis* were assigned to the 6 already known insect cytosolic GST clades, except 2 sequences assigned to two distinct unclassified clades (Un.1 and Un.2; Figure 1 and Table S2). In addition, we identified 3 microsomal sequences, that represent the highest number of MAPEGs in antennal transcriptome described so far. In *E. semipurpurella* and *C. sasakii* we also identified respectively 1 and 2 antennal MAPEGs (Table 1). Absence of MAPEG report in the other moth species could probably be due to previous incomplete annotations restricted to cytosolic GSTs.

According to our phylogenetic tree, epsilon, sigma and delta classes represent the major part of the identified sequences in *S. littoralis*, distributed into 5 well supported sub-clades for epsilon GSTs and 2 sub-clades for sigma and delta GSTs. As shown by the short branch length in the phylogenetic tree, antennal GSTz1 sequences are much conserved in moths, even between non-dytrisian and ditrysian species (90.45% of identity between *E. semipurpurella* and *S. littoralis* corresponding sequences). The ratios of non-synonymous to synonymous substitutions were estimated for 10 GSTz1 (Table S3). Their values far <1.0 indicate that these genes are under strong purifying selection pressure, suggesting a functional conservation. The selection pressure for the GSTz2 genes seems not as important with only 67.1% amino acid identity between BmorGSTz2 and SlitGSTz2 (Figure 1).

Amongst SlitGSTs from the delta class, SlitGSTd2 is of particular interest as it falls into a basal clade containing one or two sequences from every species. Moreover, all the delta GSTs from moths known to have an antennal specific expression, such as MsexGST-msolf from *M. sexta* (Robertson et al., 1999; Rogers et al., 1999), BmGSTd4 from *B. mori* (Tan et al., 2014), GST-haolf (Wang et al., 2004) from *H. armigera* and CpomGSTd2 (Huang et al., 2017) from *C. pomonella* fall within this clade.

### Tissue-related expression of *S. littoralis* GSTs

**Figure 2:**
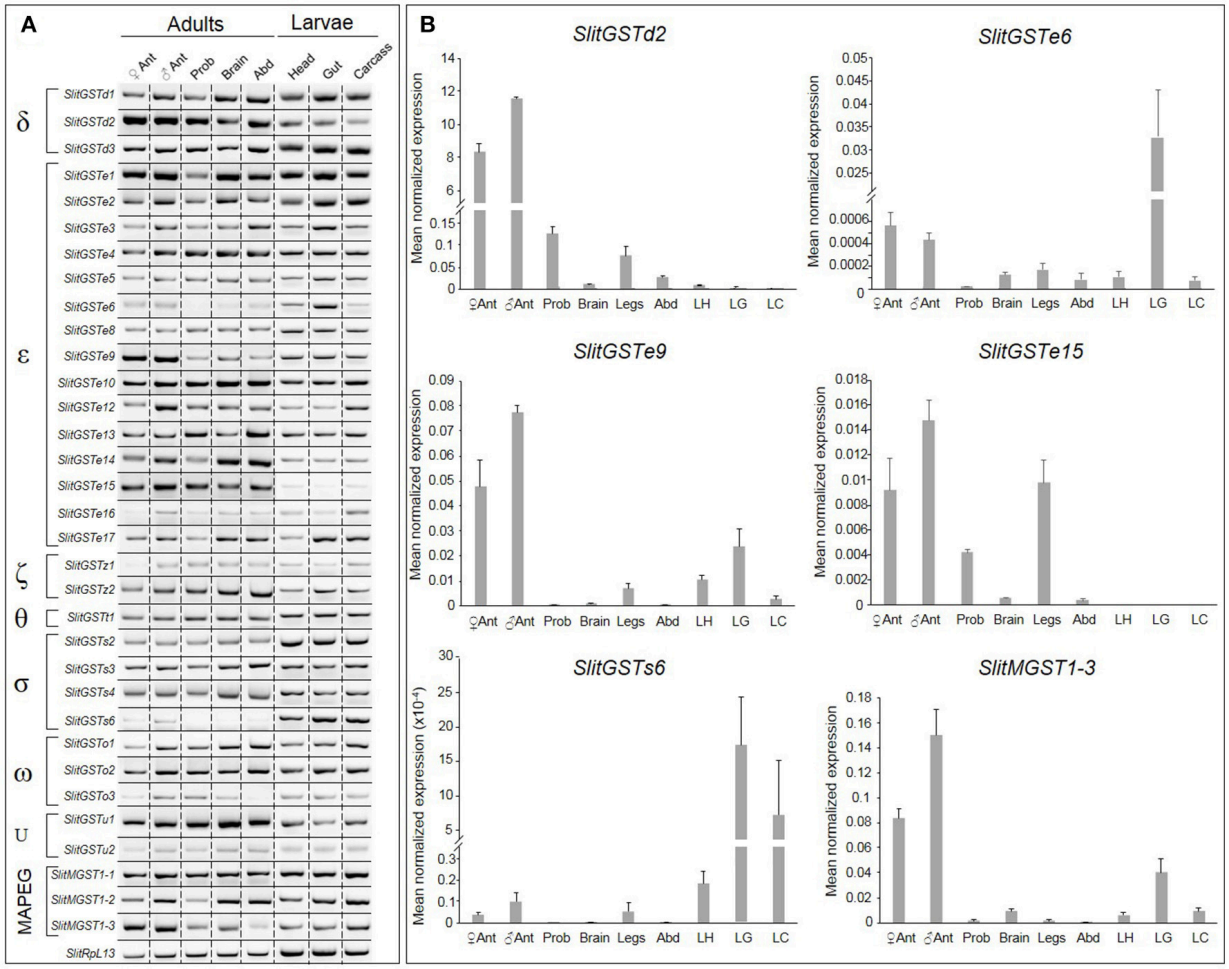
Analysis of *Spodoptera littoralis* GST expression throughout the body. **(A)** RT-PCR products visualized on agarose gel. **(B)** Expression of *SlitGSTd2, SlitGSTe6 SlitGSTe9, SlitGSTe15, SlitGSTs6*, and *SlitMGST1-3* by qPCR. Data were obtained from triplicate experiments and are given as the Mean ± SD. Templates were from adult female antennae, adult male antennae, proboscis, brains, abdomen and larval heads, guts and carcasses. *SlitRpl13* was used as the control gene.

Seven adult and larval tissues were tested in order to precise the expression patterns of *SlitGSTs* by RT-PCR and qPCR. Most of them are expressed in all the tissues examined (Figure 2A). *SlitGSTe15* is preferentially expressed in adults whereas *SlitGSTs2* and *SlitGSTs6* are preferentially expressed in larval tissues (Figures 2A,B). *SlitGSTd2, e9, e6*, and *SlitMGST1-3* seemed preferentially expressed in adult antennae, as confirmed by qPCR on the same tissues but including male legs (Figure 2B). These genes are also expressed at low level in other chemosensory or nervous adult tissues, such as in the proboscis and in the legs for *SlitGSTd2*, in the legs for *SlitGSTe9* and in the brain for *SlitMGST1-3*. All three genes seem more expressed in male antennae than in female antennae but they are not male enriched.

### Identification of predicted signal peptide in insect GST sequences

As seen previously, *SlitGSTd2* clusters in a conserved subgroup which includes *BmGSTd4* and *CpomGSTd2* for which predicted signal peptide (SP) sequences have been identified (Tan et al., 2014; Huang et al., 2017). *SlitGSTd2* possesses also a predicted 22 amino acid SP at the N-terminus of the protein sequence, and its presence was confirmed by using RT-PCR with specific primers (Figure S1). Screening of the 36 remaining *S. littoralis* GST sequences confirmed that *SlitGSTd2* was the only sequence presenting this feature.

To test whether this characteristic was shared with the other members of the subclade, we first searched for SP in the sequence of *AtraGST* (Leal et al., 2009), *MsexGST-msolf* (Robertson et al., 1999; Rogers et al., 1999), *CsupGSTd1* (Liu et al., 2015a) and *CmedGSTd1* (Liu et al., 2015b) and we indeed identified putative SPs for all them. We then completed by bioinformatics the N-terminus sequences for the 5 other genes from this clade (*SfruGSTd2, HarmGSTd02, BmGSTd1, EposGST11*, and *PxylGSTd1*) and we also identified predicted SP for each of them. All the members of the *SlitGSTd2* cluster thus contain a predicted SP.

**Figure 3:**
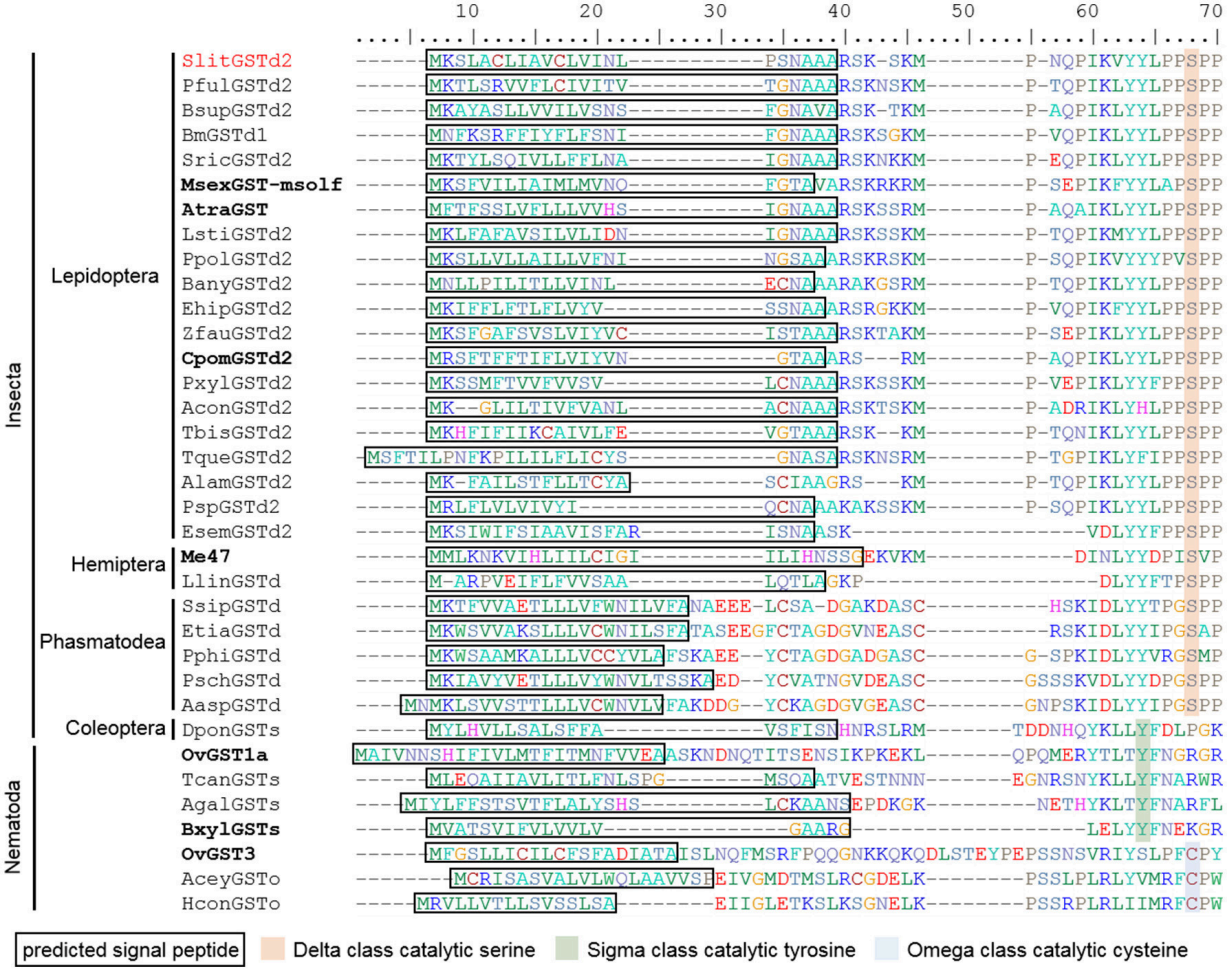
Partial alignment of 35 GSTs from insects and nematodes showing several sequences from delta, sigma and omega classes with predicted signal peptides. Each sequence is representative of one family. Slit, *Spodoptera littoralis*; Bsup, *Biston suppressaria*; Msex, *Manduca sexta*; Atra, *Amyelois transitella*; Bany, *Bicyclus anynana;* Ehip, *Eogystia hippophaecolus;* Zfau, *Zygaena fausta*; Cpom, *Cydia pomonella*; Pxyl, *Plutella xylostella*; Tbis, *Tineola bisselliella*; Tque, *Tischeria quercitella*; Alam, *Andesiana lamellata*; Psp, *Ptyssoptera* sp.; Esem, *Eriocrania semipurpurella*; Llin, *Lygus lineolaris*; Meup, *Macrosiphum euphorbiae*; Ssip, *Sipyloidea sipylus*; Pphi, *Phyllium philippinicum*; Aasp, *Aretaon asperrimus*; Psch, *Peruphasma schultei*; Dpon, *Dendroctonus ponderosae*; Ovol, *Onchocerca volvulus*; Tcan, *Toxocara canis*; Agal, *Ascaridia galli*; Bxyl, *Bursaphelenchus xylophilus*. BxylGSTs full name is BUX.s000647.112 (Espada et al., 2016a). Identifiers in bold indicate sequences linked to representative publications. Pful, *Phragmatobia fuliginosa*; Bm, *Bombyx mori*; Sric, *Samia ricini*; Lsti, *Loxostege sticticalis*; Ppol, *Papilio polytes*; Acon, *Argyresthia conjugella*; Etia, *Extatosoma tiaratum*.

We then searched the public databases and the literature for insect GSTs possessing this unusual feature and identified predicted SP in 67 full-length GST sequences (Table S4). Putative SP is present in the sequences of GSTs from the delta clade in 38 lepidopteran species, including 6 non-dytrisian species, in 6 hemipteran and 9 phasmopteran species. SP was also isolated in a sigma GST from a Coleoptera, the mountain pine beetle *Dendroctonus ponderosae*. Finally, we found 12 sigma and omega GSTs with SP in nematodes, another group of Ecdysozoa. Alignment of SP sequences from some insect and nematode GSTs (Figure 3) revealed that these signal peptides are 15–26 amino acid long and generally end with one to three alanine residues as amino acids with hydrophobic side chain. In Lepidoptera, the amino acids around the cleavage site are well conserved with a N-A-A/X-A followed by a RSK motif.

## Discussion

### Diversity of GSTs in *S. littoralis* antenna

In the cotton leafworm *S. littoralis*, an unsuspected number of CCEs, UGTs, and CYPs had been previously described in antenna (Durand et al., 2010b; Pottier et al., 2012; Bozzolan et al., 2014), leading to the hypothesis that antennal enzymes could participate in signal inactivation and odorant clearance as Odorant-Degrading Enzymes, but also in detoxification processes. Indeed, various airborne compounds, such as toxic allelochemicals emitted by plants or anthropic xenobiotics could enter the olfactory sensilla and reach the olfactory receptor neurons (ORNs) and potentially harm them (Siaussat et al., 2014). In this study, we demonstrate an unsuspected diversity of GSTs in *S. littoralis* antenna. With 33 described GSTs, the number of antennal-expressed genes is more abundant than any other Lepidopteran described so far.

Without any genome-scale information, the total number of GSTs from *S. littoralis* cannot be predicted. However, with the recent completion of the genome of *S. litura*, a sister species (47 GSTs annotated, Cheng et al., 2017), and from a closely related species *S. frugiperda* (46 GSTs, Gouin et al., 2017), we can speculate that at least two third of total GSTs could be present in *S. littoralis* antennae. This massive expansion, drived by multiple gene duplications and polymorphism, is suspected to be part of *S. littoralis* ability to detect and detoxify many plant compounds, enabling this species to cope with numerous host plants and may contribute to insecticide resistance (Elghar et al., 2005).

Phylogenetic analysis based on available antennal transcriptomes and genomes from representative lepidopterans, including non-ditrysian basal orders showed that all SlitGST sequences fall into specific clades, with notable expansions in Noctuid family. This great number of orthologous GST groups in moths suggests recent radiation/expansion events in these species.

### Microsomal antennal GSTs

In insects, the MAPEG clade contains in general few gene duplicates but seems involved in various metabolic pathways, including aging and pesticide detoxification. Indeed, a mutation in *D. melanogaster* MGST reduced lifespan, and in *Nilaparvata lugens*, GSTm2 expression level is induced by several pesticides (Toba and Aigaki, 2000; Zhou et al., 2013). If *S. littoralis* MAPEGs are very conserved, their expression pattern is remarkably different. *SlitMGST1-1* and *SlitMGST1-2* were indeed broadly expressed in all tissues and development stages tested whereas *SlitMGST1-3* was restricted to the antenna suggesting that these three genes could have evolved different functions after a noctuid-specific duplication event. A previous study showed that *SlitMGST1-3* expression is induced after deltamethrin exposure (Lalouette et al., 2016), thus suggesting a possible specialization of SlitMGST1-3 toward toxic molecules. In the beetle *P. striolata* two MGSTs are also preferentially expressed in the antennae of adult insects (Wu et al., 2016) meaning that members of this protein family could have conserved important function linked to olfaction.

### Sigma GSTs

We identified 4 sigma GSTs in *S. littoralis* antenna, which correspond to the second major expansion described in our phylogenetic analysis. Their expression pattern is similar in any tested tissue, they are highly expressed both in larval and adult tissues with the exception of *SlitGSTs6* whose expression is almost restricted in the larval midgut and carcass. Moreover, *SlitGSTs6* clustered in a conserved group with other larval specific enzymes, like *PxylGSTs1* (You et al., 2015). These results are in agreement with the already described expression patterns of other sigma GSTs whose function is often associated with xenobiotic detoxification in insect larval midgut (Huang et al., 2011; Qin et al., 2014). In particular, their expression level is affected after pesticide exposure suggesting a role in response toward toxic compounds and subsequent generated oxidative stress.

Overall, our results suggest that sigma GSTs could be separate in two clades: one with a broad expression, including antenna, associated with basal conjugation functions and one larval-specific, which activity could be linked with oxidative stress response.

### Omega GSTs

Omega GSTs define a particular clade as their catalytic properties differ from other GSTs. Indeed, GSTo have a specific dehydroascorbate reductase and thiol transferase activities conferring an oxidative stress protection (Yamamoto et al., 2009a). This conserved function is associated with an apparent 1:1 orthology relationship of the three GSTo groups identified in our phylogenetic tree, with exception of *B. mori* (4 GSTo) and *P. xylostella* (5 GSTo). Moreover, expression analysis confirms a consistent pattern throughout any conditions. A GSTo in the silkworm is highly expressed in an insecticide-resistant strain and shows high affinity with organophosphate insecticides, indicating that it may contribute to insecticide resistance and oxidative stress responses, a potential conserved role across lepidopteran GSTs (Yamamoto et al., 2011).

### Zeta and theta GSTs

The role of the zeta class GSTs had been first linked with phenylalanine and tyrosine catabolism, as maleylacetoacetate isomerases, suggesting a constitutive expression during every life stages (Board et al., 1997). However, *BmGSTz* had been associated with permethrin resistance with a predominant expression in fat body (Yamamoto et al., 2009b). This two opposite results illustrate the potential role of the two GSTz identified in Lepidoptera, which defined two highly supported clades with a strict 1:1 orthology between species. Thus, we can speculate about their function, with extremely conserved zeta1 GSTs as maleylacetoacetate isomerases (under strong purifying selection pressure) and zeta2 GSTs involved in insecticide resistance. We observed a ubiquitous expression of *SlitGSTz1* and *z2*, in agreement with their proposed functions.

As expected, we also identified a single theta GST in *S. littoralis*, widely distributed in various tested tissues. In *B. mori*, GSTt1 has been shown to possess a role in defense mechanisms against oxidative stress and in the metabolism of lipid peroxidation products (Yamamoto et al., 2005).

### Unclassified GSTs

According to our phylogeny, unclassified GSTs segregate in two paralogs groups, each composed of 1:1 orthologs from each lepidopteran species. Functional information regarding those clades is scarce. *L. migratoria GSTu1* is expressed in Malpighian tubules and its downregulation using RNAi leads to a higher sensitivity to carbaryl and chlorpyrifos insecticides (Qin et al., 2013). In silkworm, *BmGSTu2* is induced in a resistant strain and is able to conjugate glutathione to the organophosphate insecticide diazinion (Yamamoto and Yamada, 2016). *SlitGSTu1* and *u2* are found to be expressed both at larval and adult stages, in all tissues tested, with a predominant expression of *SlitGSTu1*. It is likely that those genes could share similar functions than the one observed in other insects.

### Epsilon and delta GSTs

Epsilon and delta clades are the most common GSTs in insects. They are widely recognized to have specific detoxification functions related to resistance to various insecticides (Enayati et al., 2005). In *S. littoralis*, epsilon clade accounts for almost half of the described sequences, with various expression patterns ranging from ubiquitous to antennal enriched genes. SlitGSTe clustered with lepidopteran GSTe functionally involved in insecticide conjugation and protection against oxidative stress in *B. mori, H. armigera, S. litura*, and *S. exigua* (Huang et al., 2011; Yamamoto et al., 2013; Liu et al., 2015b; Xu et al., 2015; You et al., 2015; Zhou et al., 2015; Wan et al., 2016; Zhang et al., 2016; Hirowatari et al., 2017; Labade et al., 2018). Moreover, three *P. xylostella* GSTe are also preferentially expressed in the antennae (He et al., 2017), suggesting a potential role in olfaction. *SlitGSTe9* and *SlitGSTe6* were predominantly expressed in the adult antenna, legs and larval midgut; in addition, *SlitGSTe15* was restricted to adult life stages and restricted to chemosensory tissues. This expression pattern suggests a possible role in olfaction and gustation aside from classical detoxification processes encountered in larval midgut.

Of particular interest is a clade containing single sequences from each lepidopteran species where *SlitGSTe16* appeared as a putative ortholog of *B. mori noperra-bo* (*BmorGSTe7*), a GST with cholesterol transporter activity involved in ecdysteroid biosynthesis (Enya et al., 2015). As *SlitGSTe16* is expressed in all tissues, a potential role in endocrine plasticity could be mediated by this enzyme, including in antennae, as ecdysteroids have been shown to modulate *S. littoralis* olfactory response (Bigot et al., 2012).

Delta GSTs sit in two conserved clades; the first one with *SlitGSTd1/d3* includes enzymes capable to metabolize pesticides in *B. mori* and *H. armigera* (Yamamoto et al., 2012; Labade et al., 2018) and exhibiting ubiquitous expression pattern. However, as far as expression information is available, the second one with *SlitGSTd2* is only composed of antennal-specific enzymes, such as *B. mori, M. sexta*, and *A. transitella* and *C. pomonella* GSTs (Rogers et al., 1999; Leal et al., 2009; Tan et al., 2014; Huang et al., 2017). MsexGST-msolf has been shown to degrade odorants *in vitro* and may have a role of ODE, especially toward aldehyde odorants, whereas CpomGSTd2 is active toward insecticides. SlitGSTd2 and the other GSTs from this clade may have evolved a function in odorant degradation and/or in protection of the ORNs toward toxic molecules in moth antennae. According to our analysis, *SlitGSTd2* is overexpressed in antenna and has the highest expression level, compared to any other genes tested here. Such an expression pattern in consistent with an ODE function as olfaction associated proteins, like OBPs, use to be highly expressed in antenna and to have a rapid turnover in this tissue (Leal, 2013).

In addition, our bioinformatics analysis revealed that all the delta-2-like GST sequences from this clade possess a signal peptide signature, suggesting that they may be secreted proteins. More globally, our extensive analysis of signal peptide presence in GSTs from various insect orders revealed that this structural feature is more widely spread than suspected and not restricted to delta GSTs, as we found also a SP in an antennal GST sigma from a coleopteran species. Extracellular GSTs have been previously characterized in several Nematodes species (Sommer et al., 2001; Liebau et al., 2008; Espada et al., 2016b), and in the pine wood nematode *Bursaphelenchus xylophilus*; one of these enzyme metabolize various terpenoid compounds (Espada et al., 2016a), also known as common odorant molecules for insects. These antennal GSTs could thus be secreted in the sensillar lymph surrounding the sensory neurons, where they would directly interact with their relative substrate. Several mode of action are likely to occur in this aqueous environment: GSTs can conjugate their substrate with glutathione, as demonstrated in vertebrates where both GSTs and GSH are found in the olfactory mucus (Krishna et al., 1992; Debat et al., 2007); alternatively the binding properties of GSTs (as ligandins, Gonzalez et al., 2018) could act as a scavenger of odorants and harmful compounds.

We have revealed in this present work the occurrence of a high diversity of GST genes expressed in the olfactory organ of a pest moth. Phylogenetic analysis showed that these genes were distributed amongst the well-defined insect GSTs clades, in agreement with different cellular localization. The SlitGST structural diversity together with their different relative spatial and developmental expression probably reflects their functional divergence and substrate specificities. Amongst this large repertoire, antennal GSTs could play a dual function in this tissue; first as detoxifying enzymes, where they could protect this delicate organ toward harmful compounds, but they could also play a role in the dynamic of olfactory signal: the conjugation of odorants (or their relative metabolites) could induce the termination of olfactory signaling. Moreover, the conjugation of such molecules may play a crucial role in odorant clearance, with the removal of any olfactory-active compounds in the sensillar lymph. Of particular interest is SlitGSTd2, as it is antennal specific, probably secreted and likely to be involved in dual mechanisms in olfactory together with detoxification functions. Further studies using *in vitro* biochemical assays will reveal SliGSTd2 function and substrate specificity, and will decipher if this enzyme is more related to toxic compounds, odorants or both. Overall future characterization, in particular biochemically but also physiologically, will allow to understand the precise function of all this enzymatic diversity in such a specialized organ, and to unravel their precise role in insect's biology as xenobiotic metabolizing enzymes and/or odorant degrading enzymes.

## Author contributions

ND, FB, and TC performed analysis. M-AP and DS provide material. ND, MM, and TC wrote the manuscript.

### Conflict of interest statement

The authors declare that the research was conducted in the absence of any commercial or financial relationships that could be construed as a potential conflict of interest.
