# Supplementary material for: Glutathione-S-Transferases in the Olfactory Organ of the Noctuid Moth Spodoptera littoralis, Diversity and Conservation of Chemosensory Clades
Source: Front Physiol. 2018 Sep 27;9:1283. doi: 10.3389/fphys.2018.01283 (PMC6171564; doi:10.3389/fphys.2018.01283)
Supplement: Supplementary file 2 [file Table_2.DOCX]

**Figure S4.** GST with SP full alignment

10 20 30 40 50 60 70 80 90 100

....|....|....|....|....|....|....|....|....|....|....|....|....|....|....|....|....|....|....|....|

SlitGSTd2 ------MKSLACLIAVCLVINL-----------PSNAAARSK-SKM--------P-NQPIKVYYLPPSPPCRSIIMAAKVIGVDLDLVLTNIMEGHHMTP

PfulGSTd2 ------MKTLSRVVFLCIVITV-----------TGNAAARSKNSKM--------P-TQPIKLYYLPPSPPCRAVMMTAKVLSLDLELIPTNVLEGAHMTP

BsupGSTd2 ------MKAYASLLVVILVSNS-----------FGNAVARSK-TKM--------P-AQPIKLYYLPPSPPSRAVMMTARAVGLDLDLVTTNIMNGEHMTP

BmGSTd1 ------MNFKSRFFIYFLFSNI-----------FGNAAARSKSGKM--------P-VQPIKLYYLPPSPPCRAVMMTARVLELDLHLITTNIMNGEHMTP

SricGSTd2 ------MKTYLSQIVLLFFLNA-----------IGNAAARSKNKKM--------P-EQPIKLYYLPPSPPCRSVMMTAEALGIQLDLISTNVMNGDHLTP

MsexGST-msolf ------MKSFVILIAIMLMVNQ-----------FGTAVARSKRKRM--------P-SEPIKFYYLAPSPPCRAVMMAARALDLELDLIPTNIMDGDHKTP

AtraGST ------MFTFSSLVFLLLVVHS-----------IGNAAARSKSSRM--------P-AQAIKLYYLPPSPPCRAVMMTAKAIGLDLNMVLTNIMEGAHMTP

LstiGSTd2 ------MKLFAFAVSILVLIDN-----------IGNAAARSKSSKM--------P-TQPIKMYYLPPSPPCRAVLMAARVLGVELDLVVTNLLEGEHLSP

PpolGSTd2 ------MKSLLVLLAILLVFNI-----------NGSAAARSKRSKM--------P-SQPIKVYYYPVSPPCRSVLLTARLLGLKVELVLINIMEGEQKSP

BanyGSTd2 ------MNLLPILITLLVINL------------ECNAAARAKGSRM--------P-TQPIKLYYLPPSPPCRSVMMTARALDIDLELVLTNIMQGEHKTP

EhipGSTd2 ------MKIFFLFTLFLVYV-------------SSNAAARSRGKKM--------P-VQPIKFYYLPPSPPCRAVIMTARILGIDLDLIVTDIMEGQHMTP

ZfauGSTd2 ------MKSFGAFSVSLVIYVC-----------ISTAAARSKTAKM--------P-SEPIKLYYLPPSPPCRAVMMTAKALGLDLDLNMTNILEGAHMTP

CpomGSTd2 ------MRSFTFFTIFLVIYVN------------GTAAARS---RM--------P-AQPIKLYYLPPSPPCRAVMMLAKVMGLELDLEITNIMIGEHKTP

PxylGSTd2 ------MKSSMFTVVFVVSV-------------LCNAAARSKSSKM--------P-VEPIKLYYFPPSPPCRAVMMAAKAMGIELEMVLTNIIEGEHMTP

AconGSTd2 --------MKGLILTIVFVANL-----------ACNAAARSKTSKM--------P-ADRIKLYHLPPSPPCRAVRMLAMALNLELELVMTNLMEGEHLKP

TbisGSTd2 ------MKHFIFIIKCAIVLFE-----------VGTAAARSK--KM--------P-TQNIKLYYLPPSPPCRSVMMTARALGLELELNLTNVMEGAHMTP

TqueGSTd2 -MSFTILPNFKPILILFLICYS------------GNASARSKNSRM--------P-TGPIKLYFIPPSPPCRAVMMAAKALGVELELIMTNLMEGEHLKP

AlamGSTd2 -------MKFAILSTFLLTCYA-----------SCIAAGRS---KM--------P-TQPIKLYYLPPSPPCRAVMMTARALDLDLELIITNIMEGDQLKP

PspGSTd2 ------MRLFLVLVIVYI---------------QCNAAAKAKSSKM--------P-SQPIKLYYLPPSPPCRAVMMTAKVLGLELDMVLTNIMEGDNMKP

EsemGSTd2 ------MKSIWIFSIAAVISFAR----------ISNAASK-------------------VDLYYFPPSPPCRAVMMLAKAINLDLNMKLTNLMQGDHLKP

Me47 ------MMLKNKVIHLIILCIGI----------ILIHNSSGEKVKM------------DINLYYDPISVPCRAVLLTLEHLNLEFNLIYIKLLDRMQLSE

LlinGSTd -------MARPVEIFLFVVSAA-----------LQTLAGKP-------------------DLYYFTPSPPCRVVMMTAKVLGADLNLKMTDITTGDQLKP

SsipGSTd ------MKTFVVAETLLLVFWNILVFANAEEE-LCSA-DGAKDASC----------HSKIDLYYTPGSPPCRSVILAAKAVGVELNLKLTDLRAGEHLKP

EtiaGSTd ------MKWSVVAKSLLLVCWNILSFATASEEGFCTAGDGVNEASC----------RSKIDLYYIPGSAPCRSVRLAASAVGLDLNLKLTNLRAGEHLKP

PphiGSTd ------MKWSAAMKALLLVCCYVLAFSKAEE--YCTAGDGADGASC--------G-SPKIDLYYVRGSMPCQAVRLTAKAIGVDLDLKLMDLRAKEQFKP

PschGSTd ------MKIAVYVETLLLVYWNVLTSSKAED--YCVATNGVDEASC--------GSSSKVDLYYDPGSPPCRAVILAAKAVGVDLNLKLTDLRAGEHRTP

AaspGSTd ----MNMKLSVVSTTLLLVCWNVLVFAKDDG--YCKAGDGVGEASC--------GNPSKIDLYYIPGSPPCRSVLMAAKAVGVDFNLKLTKLFAGEHLTP

DponGSTs ------MYLHVLLSALSFFA-------------VSFISNHNRSLRM-------TDDNHQYKLLYFDLPGKAEHIRYIFAYAGVEYE----DERIQKDKWP

OvGST1a MAIVNNSHIFIVLMTFITMNFVVEAASKNDNQTITSENSIKPKEKL-------QPQMERYTLTYFNGRGRAEVIRLLFALANVSYE----DNRITRDEWK

TcanGSTs ------MLEQAIIAVLITLFNLSPG--------MSQAATVESTNNN-------EGNRSNYKLLYFNARWRGEGARLIFAYAGVPYE----DVRVTKEEWP

AgalGSTs ----MIYLFFSTSVTFLALYSHS----------LCKAANSEPDKGK---------NETHYKLTYFNARFLGEGPRQVLQEAGIPFE----DIRIDKKDWP

BxylGSTs ------MVATSVIFVLVVLV---------------GAARG-------------------LELYYFNEKGRAEPIRLMLHYAGTNFT----DFRFSRADWN

OvGST3 ------MFGSLLICILCFSFADIATAISLNQFMSRFPQQGNKKQKQDLSTEYPEPSSNSVRIYSLPFCPYGESVILAAYKKGIQFDI---GYINHPYQMN

AceyGSTo --------MCRISASVALVLWQLAAVVSPEIVGMDTMSLRCGDELK--------PSSLPLRLYVMRFCPWCERVLLYLSRKNASVEVVNVDLVDKPTF--

HconGSTo -----MRVLLVTLLSVSSLSAEIIG--------LETKSLKSGNELK--------PSSRPLRLIIMRFCPWCERVLLVIARKNMSVEVVNVSLSDKPKF--

110 120 130 140 150 160 170 180 190 200

....|....|....|....|....|....|....|....|....|....|....|....|....|....|....|....|....|....|....|....|

SlitGSTd2 EYLKMNPQHTIPTMDDSGFILWESRAILAYLANAY-SRDDTLYPKNPRQRAIVDQRLNFDLGTLYVRYSALYLPMLFRGEEYDEQKA--DQLDEA-----

PfulGSTd2 EYTKMNPQHTIPTMDDSGFILWESRAIMAYLVNAY-GRDDSLYPKNPRQRAIVDQRLNFDIGTLFARYAGLYIPMLFRGEEYNEENA--EKLNEA-----

BsupGSTd EYLKMNPQHTIPTIDDGGFILWESRAIMPYLVNAY-GKDDSLYPKNPRQRALVDQRLNFDLGTLFQRYANLYVPMLFRGDPYNEANA--AKLDEA-----

BmGSTd1 EYLKMNPQHTIPTMDDNGFILWESRAIQTYLVNAY-GKDDSLYPKNPRQRAIIDQRLNFDLGTLYLRYLNLYTPILFRGEAYDQEKA--DKFDEA-----

SricGSTd2 EFLKMNPQHTIPTMDDNGFFLWESRAIMAYLVNAY-GKDDSLYPKNPRLRAIVDQRLNFDLGTFFSRYLDLYMPILFHGEAYDETKA--EKLNEA-----

MsexGST-msolf EYLKMNPQHTIPTMDDSGFILWESRAILAYLVNAY-GKDDSLYPKNPRQRAIVDQRLNFDIGTLFPRYSNLYFPMLFRGDEYNQENA--DKLNEA-----

AtraGST EFLKMNPQHTIPTMDDNGFILWESRAIMAYLVNAY-GRDDSLYPKNPRLRAVVDQRLNFDIGTLFARYLGVYLPVLFKGEEMSQEAV--DKLNEA-----

LstiGSTd2 EFVKMNPQHTIPTMDDSGFILWESRAILTYLANAY-GKDDSLYPKNPRQRAVVDQRLNFDLGTLFMRYINLYGPMIFTGAPMDEEKA--TKLNEA-----

PpolGSTd2 EFIKMNPQHTVPTIDDNGFILWESRAIMAYLVNAY-GKDDALYPNDPQQRAIVDQRLYFDF-SFLQKSLELYWPMV-NGKEYNEEKG--EKLKEA-----

BanyGSTd2 EYLKMNPQHTIPTMDDSGFILWESRAILTYLVNAY-GRTDSLYPKNPRQRALVDQRLNFDLGTLYKRYMDLYAPMLFHGQGYNDEAA--EKLNEA-----

EhipGSTd2 EYLKMNPQHTIPTMDDNGFILWESRAIMTYLVSAY-GSDDSLYPKNARLKALVDQRLYFDLGTLYQRYLDLYAPILFKGEEYDDEKA--DKLNEA-----

ZfauGSTd2 EFLKMNPQHTIPTMDDSGFILWESRAIQAYLVNAY-GKDDSLYPKNPRLRAIVDQRLNFDLGTLYLRYITLYSPIIFRGEEYNEENA--AKLNEA-----

CpomGSTd2 EFLKMNPQHTIPTMDDNGFILWESRAIMAYLVNAY-GRDDSLYPKNPRLRALVDARLNFDLGTLFLRYLNLYAPMLFHGEEYNEESA--AKLDEA-----

PxylGSTd2 EFLKMNPQHTIPTIDDNGFILWESRAIIQYLANAY-GRDDSLYPKNPRLRAMGDQRLNFDLGTLVSRYLNLYSPVL-HGEPFSDDMD--AKLKEA-----

AconGSTd2 EFLKMNPQHTLPTIDDNGFILWESRAIMAYLVNAY-GRDDSLYPKNPRLRAVVDQRLNFDVGTLYARYIAYYAPVLFFGQEKDEEKQ--KKLDEA-----

TbisGSTd2 EFLKINPQHTIPTIDDNGFILWESRAILAYLVNAY-GKDDSLYPKNPRLRALVDQRLNFDLGTLYPRYFNLYMPMIFRGAKYDNELA--DKLNEA-----

TqueGSTd2 EFIKMNPQHTLPTIDDNGFILWESRAIMQYFANAY-GRDESLYPKNPRLRALVDQRLQFDQGTLFPRYFDVYHTIIANGGEIDEAKA--DKLREA-----

AlamGSTd2 EFIKMNPQHTIPTMDDSGFYLWESRAIMAYLVNAY-GKNDSLYPKNPRLRALVDQRLNFDLGTLYSRYITAYVPVIFHGEEMDEDSA--KNLSDA-----

PspGSTd2 EFIKMNPQHTIPTMDDSGFYLWESRAIMSYLVSAY-GRDDSLYPKNPRLRAIVDQRLNFDLGTLYSRYLALYGQAIFTGASLDEANI--EKMHEA-----

EsemGSTd2 DFIKMNPLHTIPTIVDNGLIMWESRAIMAYFVNQY-GPDDSLYPKDPKMRAIIDHRLNFDLGTLAARFITYYIPTVFAGAPLDPEKL--KPLEEA-----

Me47 DFRKINPLHTIPVLEDGDFVLSDSHAIIIYLVREYGGKDNSLYPNDPKFQAQVHQRLFFDDSTLYMAFRLEYNPRIYYRTPKTEKGE--EKVQNA-----

LlinGSTd EFLKINPQHTVPTFDDNGFVLSESRAIAAYLAETS-PNGEKIYPKDPKKRALIEQMLYFDIGRLYQNFLDLYKPML-KGKPFDDALG--KTFDES-----

SsipGSTd EFLKMNPQHKVPTINDNGFLLGESRAIMGYLVDKY-AKDDSLYPKDPAKRALVNQRLYFDIGTLYNAFSEFYYPMAFSGASADPEKK--KKLENA-----

EtiaGSTd DFIKMNPQHKVPTINDNGFLLGESRAIMGYLVDQY-AKDDTLYPKDPKKRAVVNQRLYFDIGTVYQRFSEYYYPLAFEGASPDPKKM--KNLEDA-----

PphiGSTd EFIKMNPQHKVPTINDNGFSLGESRAIMGYLVDQY-AKNDDLYPKNAKKRALVNQRLYFDIGTLYQRFSDYYYPLAFEGAVPDKNKL--EKFEEA-----

PschGSTd EFLKMNPQHTVPTINDNGFFLAESRAIMGYLVDQY-AKDDALYPKNAKERALVNQRLFFDIGTLYQRFSDYYYPIAFGGASPDKEKM--KKLEEA-----

AaspGSTd EFLKMNPQHIVPTIDDNGFILAESRAIMGYLVDQY-AKDDTLYPKNAKKRALVNQRLYFDIGTLYQRFSDYYYPIGFGGAPPDPKKM--EKLEEA-----

DponGSTs EIKKSTPYGKVPVLEIDGQQVAQSNAIARYLARKF-GLVGQTEWEALQCDVLVDT-----LGDLQAAVMQ-----IMKEPDPIKREEIRARVTKEELPFY

OvGST1a YLKPMTPFGHVPILNVSGNVLGESHAIELLLGGRF-GLLGTNAWEEAKIMAVVLN-----IEELFQKLIPWTH-----EKNTTKKAELFRNLSESNVMPF

TcanGSTs KLKPTTPFGHLPVLKFGGHELGESFAINRYLARQF-GLAGNGSLEEAYVDSIADF-----FKDFFEKTGESVKAIITGGGPIDPAY---NEIVAPARDAF

AgalGSTs ALKPKTPFGQLPILEFDGNVIAQSFAICRYLARKY-GLAGKTPLEEALVDSIADA-----EKDFFTTARPYFD--ILGGHKEGDKEAVYKEVVIPARDNF

BxylGSTs TSKDSMLFHTIPALVQDSDLLENSEAITRYVAQTA-GLLTASYIEDAQLDSLFEQ-----SGIVLRNLLEYIS--IVEGRKPGNATNARYRLVEYPCQLY

OvGST3 WFLAKNPEGALPAVEHNGELVIDSLVIMEYLDDVF--SENSILPDEPYLRAKQRY-----EAIKLDSICDAIRKVSYSKKLTGNITMLTMELTEA-----

AceyGSTo -LFSKHPEGKVPVLEHKGQNIIDSALISEYLDWIQ--PHTSILPSDPYLKA--KQRML--SGLLEGKLPAASRAIVEEQKKHSQKSLTNAAVHDA-----

HconGSTo -LLEKHPDGRVPVLMHNNKTIIDSAVIAEYL-DTL-DPAKPILPTDPDLRSKQKKM----AAKLEAQLPSAVHALINEQRFHTEKEPTIKKLHDA-----

210 220 230 240 250 260 270 280 290 300

....|....|....|....|....|....|....|....|....|....|....|....|....|....|....|....|....|....|....|....|

SlitGSTd2 LGWLNTFLDG--RAFVAGDNLTIADISIIVT------------ITNL--------EAFG-YD-M-SGHPNLTKWFERT--KKALEP--------------

PfulGSTd2 LGWLNTFLDG--RAFVAGDNLTIADISIIVT------------ITNI--------EAFG-FD-F-SQHENVKKWFERT--KKALEP--------------

BsupGSTd2 LGWLNTFLEG--RAFVAGDNLTIADISIIVT------------ITNI--------DAFG-YD-F-SAHENVTKWFERT--KKALEP--------------

BmGSTd1 LGWLNTFLDG--RPFVAGENMTVADITIVVT------------ITNI--------DAFG-YD-F-SSHENIAKWFERT--KKMLEP--------------

SricGSTd2 FGWLNTFLDG--HDFVAGENLTIADISIIVT------------VTNL--------EVFG-YD-Y-SAHENVKNWFERT--KKALEP--------------

MsexGST-msolf LGWLNIFLEK--SAFVAGDNLTIADISIIVT------------ITNL--------DAFK-FD-F-SEHENLTKWFERT--KKALEP--------------

AtraGST LGWLNTMIEG--KTFVAGDNLTIADIAIVVT------------FTNL--------EAFE-YD-F-SAYENLTQWFART--KKALEP--------------

LstiGSTd2 IGWLNTMLEG--KAFVAGDNLTLADVSIIVT------------FTNL--------EAFE-YD-F-SQYENVVKWFGNT--KKALEP--------------

PpolGSTd2 VGWLNSMLEG--RQFVAGDKLSIADITTVVT------------MSNL--------DSFG-FD-Y-SSYGNIKPWFERT--VKELEP--------------

BanyGSTd2 LGWLNSMLEG--RVFVAGDTLTIADISIVVV------------FSNL--------EAFG-FD-F-SAHENVAKWFERT--KKSLEP--------------

EhipGSTd2 LDWLNTMLEG--KTFVAGDNITIADISIVVT------------FTNL--------EALD-YD-L-NAYDNVRKWFART--KKALEP--------------

ZfauGSTd2 LGWLNTMIEG--KAFVAGDNLTIADISIIVT------------FTNL--------DALG-FD-F-SAFENITKWFERT--KKALEP--------------

CpomGSTd2 LGWLNSMLEG--RAFVAGDNMTIADITIVVT------------LTNL--------DALS-YD-F-SAHDNVTKWFERM--KKALEP--------------

PxylGSTd2 LGWFNTMLEG--RAFSAGDNLTVADISIVVV------------FSNL--------EAFG-YD-F-TAYDNVSKWFERT--KKALEP--------------

AconGSTd2 IGWFNSMLEG--RTFSAGDNLTIADITIIVT------------FSTL--------EALE-YD-F-SEYENVQKWYERT--KKALEP--------------

TbisGSTd2 LGWLDQMLEG--KVFVAGDNLTIADFSMIVV------------ITGL--------DAFG-HD-L-SPYDNVMNWYERT--KKVLEP--------------

TqueGSTd2 LGYLNTMIEG--KPFVAGENLTIADITIIVT------------ITSL--------DIFG-FD-M-SSYDNVVNWYERT--KKVMEP--------------

AlamGSTd2 LAWLEAMLDG--RAFVAGDNFTIADISIIIT------------ITNL--------SELG-YD-L-SSYENVTNWFERT--KKVLEP--------------

PspGSTd2 LDWLNTIIEG--KPFVAGDNMTIADITMVVT------------FTNI--------NAFG-FD-F-SKYENISQWFERT--KKALEP--------------

EsemGSTd2 LNILNIMLDG--SKFAAGDKLSIADFSIITT------------ISNM--------DAFG-YD-F-SPYPNVKKWYDMT--KTALKP--------------

Me47 LKFLENVLTK--SIWAAGDSMTVADFSLVSS------------ISTF--------EVAGAID-F-DKYPNIKKWFINC--KSKME---------------

LlinGSTd LAMFEEYLTR--TQWAAGDQMSIADLSLMAT------------VTTA--------EAVG-HD-F-SKYPKIKQWMDKT--KSAIP---------------

SsipGSTd FEVLDKFLEG--QKWVAGDSMTIADISLTSS------------VATP--------VAVG-FD-L-AKYKNVAKWFERC--QKSIH---------------

EtiaGSTd FGVLDKFLEG--QEWVAGDSMTIADISLTSS------------VSSP--------VVLG-FD-L-SKFKNVARWFNSC--KKSIP---------------

PphiGSTd LGVLDKFLEG--QDWVAGNSMTIADISITSS------------VSAP--------AALG-YD-L-KKFKNVAKWFDKC--KKSIP---------------

PschGSTd FGFLDKFLEG--QDWVAGSSMTIADIAITST------------VASP--------VAVG-FE-L-QKFKNVERWFGNC--KNAIP---------------

AaspGSTd FGFLDKFLDG--HEWVAGNSMTIADVSLTST------------VASA--------DVLG-FD-L-GKFKNVERWFDMS--KKSIP---------------

DponGSTs LSKFEKIVQEN-GGFSVGSDITWSDLVFAVL------------LDQF--------ESMYGKTAL-NGYPSLKGLKDKV--HNIPGI--------------

OvGST1a LGRYEKFLKESTTGHIVGNKVSVADLTVFNM------------LMTL--------DDE--VK-L-EEYPQLASFVNKI--GQMPGI--------------

TcanGSTs LPPLERFLKKANSGYVVGNSATWADLIITEY------------LATC--------QSAI-PGFL-SGYPSVESYVNRI--RLLSNI--------------

AgalGSTs LNSLEKFLEKG-CGFVVGDSLTWADLIIAEY------------LATW--------TSIK-PEFL-DGHPKVAEYVKQI--QELPNI--------------

BxylGSTs YPLIEQHIFND-TGFFGRYGITYFDFVWFNL------------SDLI--------NSYA-FDVL-AKYPVSSNHWWNVMGYNNSNL--------------

OvGST3 ----EQMLE---SPFYSGETFGLPDIVLYPC------------IQRLYMIGQTINDSFL-HNYFPDHFPKLSEWFTRM--QTLREI--------------

AceyGSTo LDSAEKLLN---STFFNGEDPGFADYMTYPFLERIWIWSHEPGATDL--------RSDA-FP-S-DAYPKLQRWFSKM--RSRKEVARVSQPLWRHRLFN

HconGSTo LDLAEKLLPN--STFYAGREPGFADYMTYPFVERIWIWSHEPGVTDL--------PADG-FP-G-ASYPKLQRWFSLM--RSTAEVKAVSQPVWRHRMFN

310 320 330

....|....|....|....|....|....|....|.

SlitGSTd2 YGYEDVDV-AGAKMLANFLKKD--------------

PfulGSTd2 YGYNEIDM-AGAQILASFLKKG--------------

BsupGSTd2 YGYNEIDA-AGAQILASFMKKE--------------

BmGSTd1 YGYDEIDV-TGAKMLASFLKKE--------------

SricGSTd2 YGYEEIDK-AGADILASFLKKE--------------

MsexGST-msolf YDWEDIDE-TGAQMLADFLKREH-------------

AtraGST YGYMEIDH-AGGQILASFIKKD--------------

LstiGSTd2 YGYEEIDV-AGAQMLAQFLKSHSS------------

PpolGSTd2 YGYVEINK-SYADKLASMIKKE--------------

BanyGSTd2 YGYEEIDQ-AGARMLASFLKKE--------------

EhipGSTd2 YGYKEIDQ-SGAQILASLLKKN--------------

ZfauGSTd2 FGYKEIDY-AGGQVLASFVNKQ--------------

CpomGSTd2 YGYKDIDY-EGAQVLASFIKKN--------------

PxylGSTd2 HGYKEIDQ-AGAQMLASFIKKD--------------

AconGSTd2 YGYKEIDE-AGAQMLGSFVKKD--------------

TbisGSTd2 FGYSEIDE-AGAKALASMLHKDN-------------

TqueGSTd2 YGQKDINE-AGAEILKEFLEKKMSG-----------

AlamGSTd2 YGYSEIDL-AGAKILASFAEKNKN------------

PspGSTd2 YGYMDIDY-AGAQILLNVMKSH--------------

EsemGSTd2 FGYEEINQ-AGANMLGDSFKANNKG-----------

Me47 -GYGKANQ-PGVDATVELLNALDVKFGKLAETPHII

LlinGSTd -DYQMANQ-DGVEIWKSMFANVKKN-----------

SsipGSTd -EYHKVSD-EGLSNFKKLFENVKTEL----------

EtiaGSTd -DYEDIAH-EDLLQFKKLFEKVKLT-----------

PphiGSTd -EYQETAH-EDTLRLKKMFENVKLN-----------

PschGSTd -EYEEITS-KGLSVFKKYFDNVKLS-----------

AaspGSTd -EYEDVTK-KGLADFRKIFGDAKLK-----------

DponGSTs KSYLEKRP-PKSPMPRH-------------------

OvGST1a KEWIKKRP-KTYF-----------------------

TcanGSTs KKWIDNRP-QSDH-----------------------

AgalGSTs KKWIEERP-KTPF-----------------------

BxylGSTs QNYFQTR---GDDFLRNETFTIYNYFNATQ------

OvGST3 QAMQEIKQYLGNFVYGSGYYNA--------------

AceyGSTo KGYIKGEP-DFDAGVGSARSRSRSV-----------

HconGSTo QGYVHGNP-DYDAGMGIRRHD---------------
