# Supplementary figures and images for: Glutathione-S-Transferases in the Olfactory Organ of the Noctuid Moth Spodoptera littoralis, Diversity and Conservation of Chemosensory Clades
Source: Front Physiol. 2018 Sep 27;9:1283. doi: 10.3389/fphys.2018.01283 (PMC6171564; doi:10.3389/fphys.2018.01283)

**Fig S1.** *SIGSTd2* full ORF

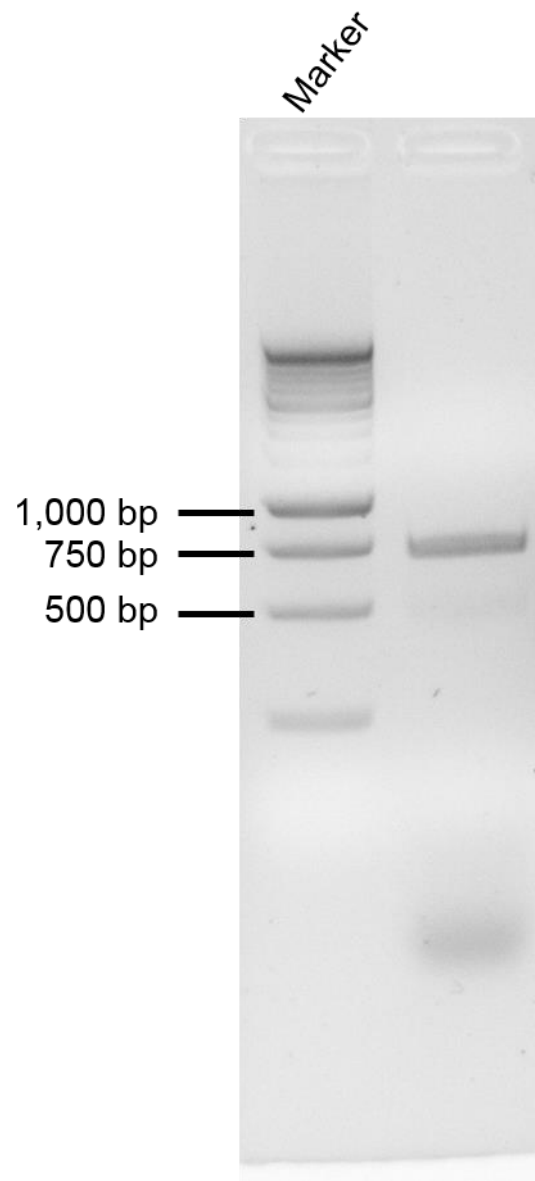

**Fig. S2.** Uncropped gels

Adult PCR gels

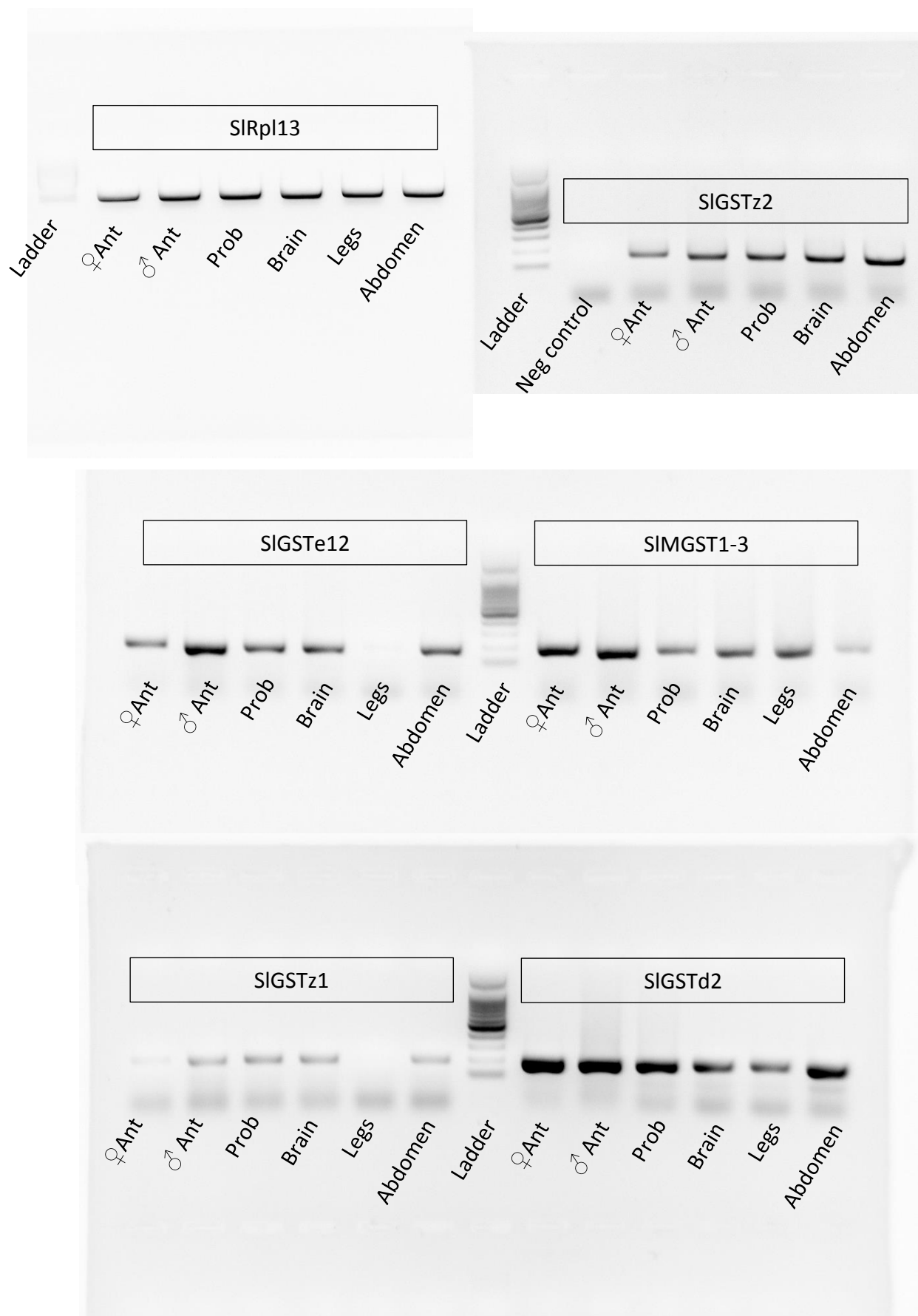

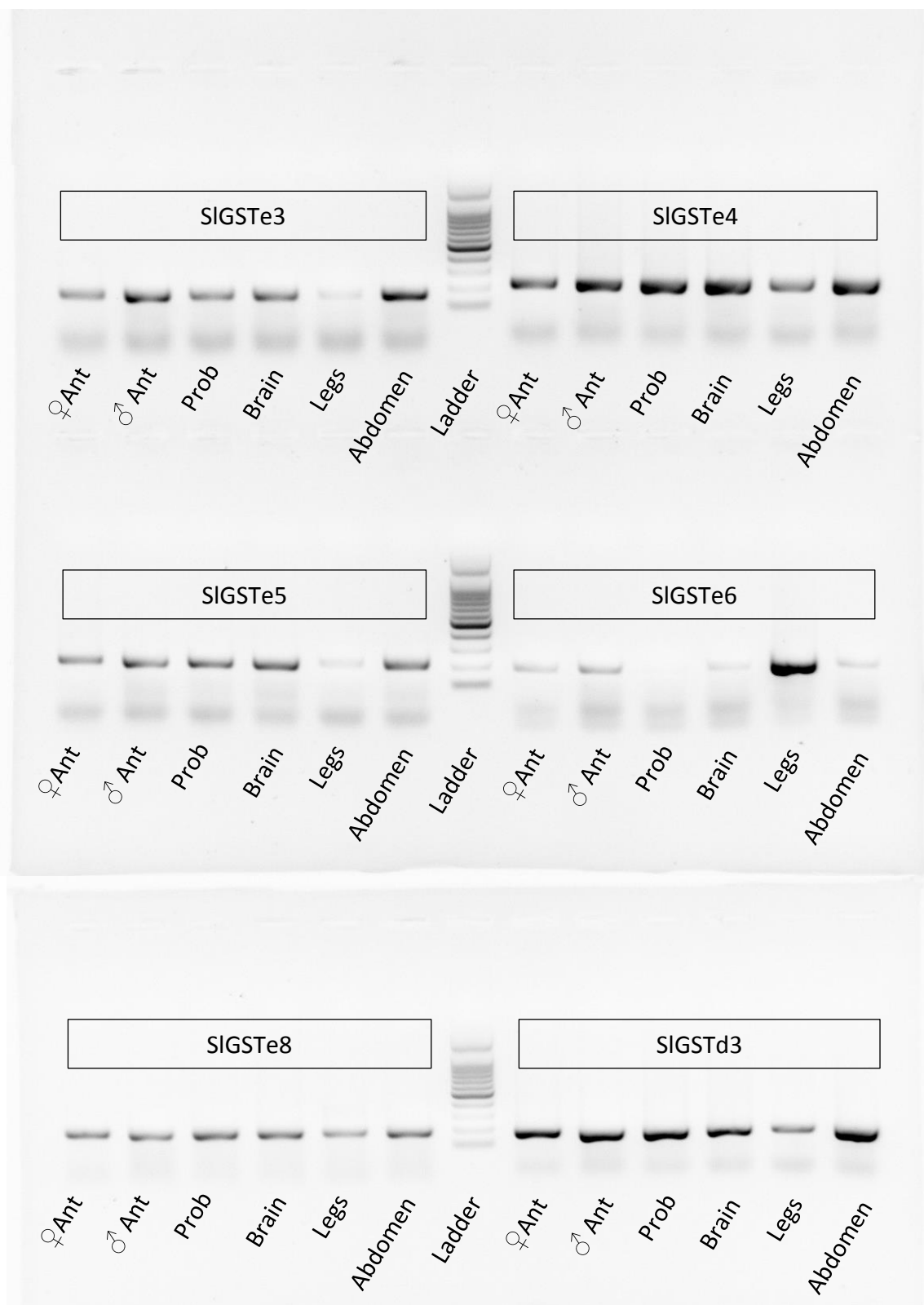

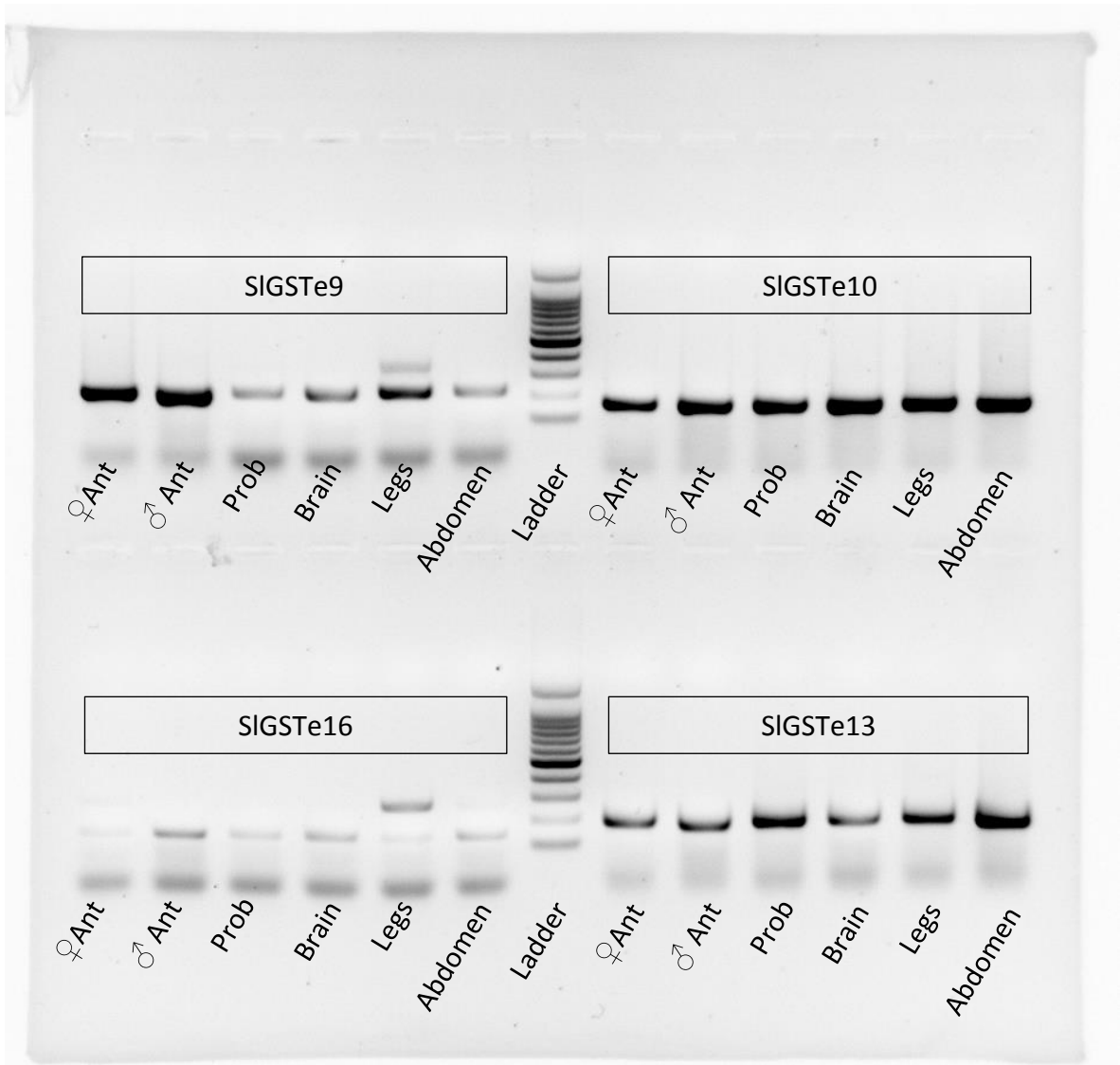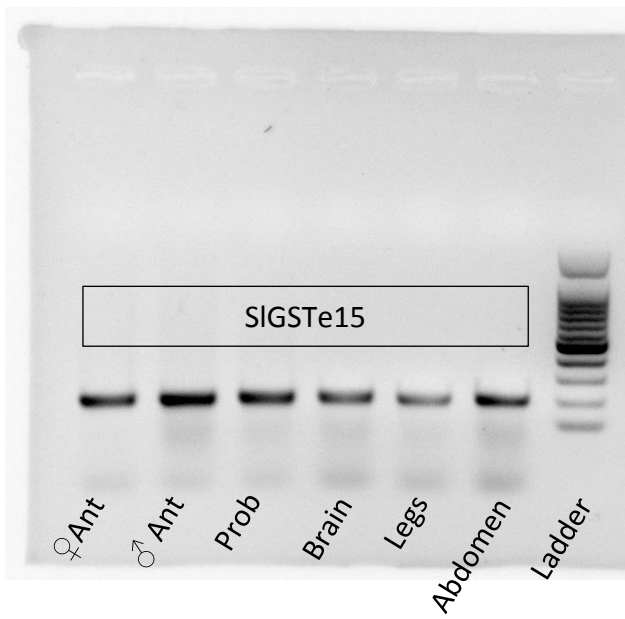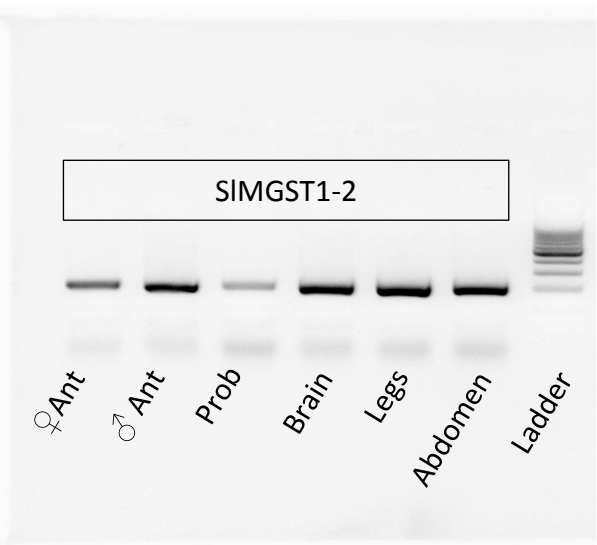

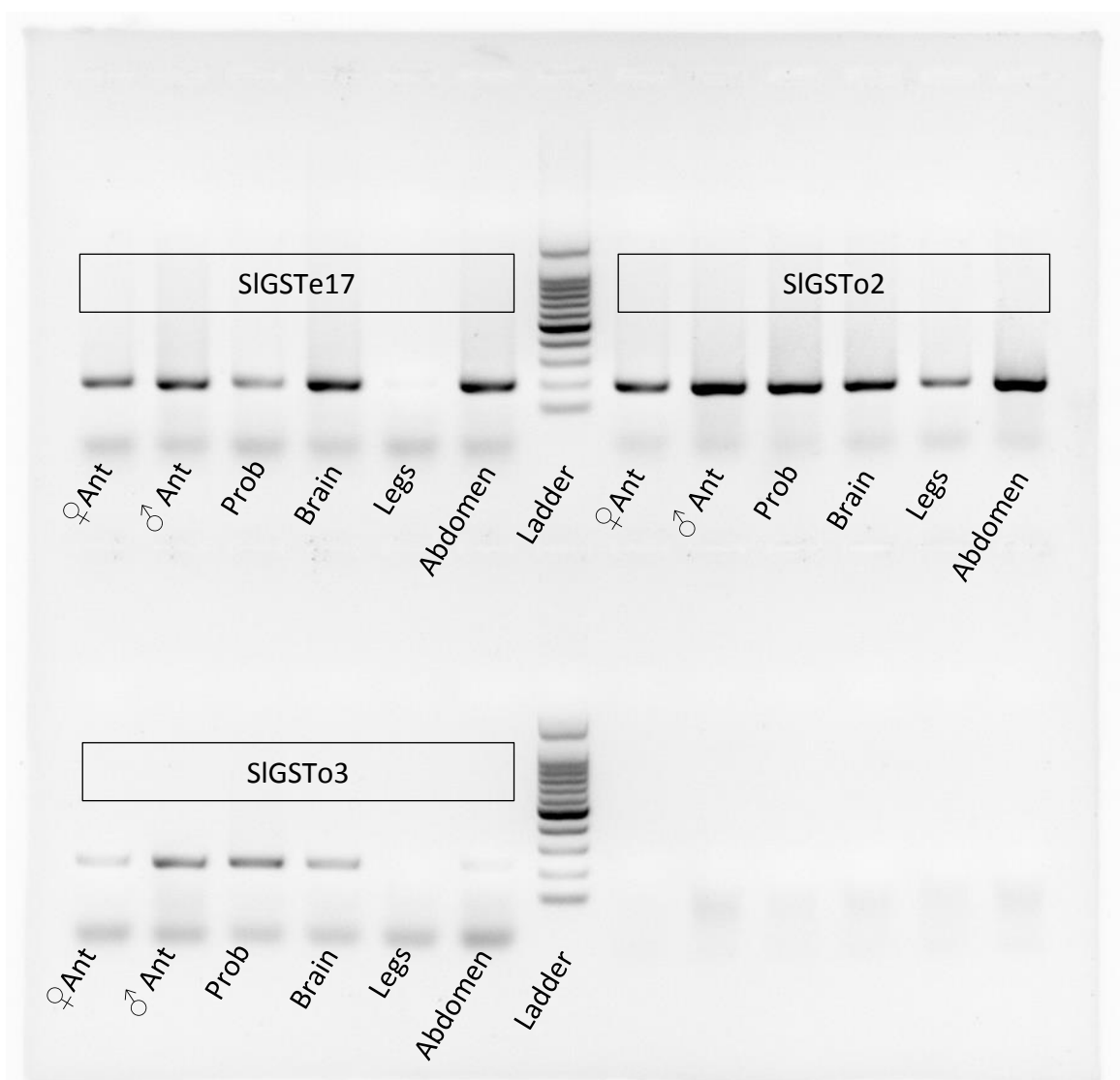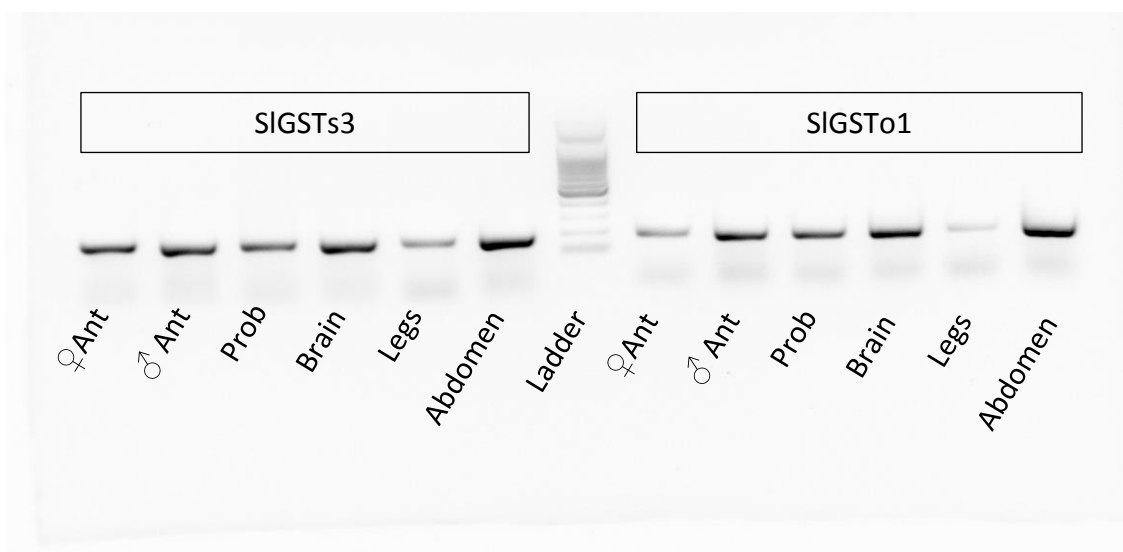

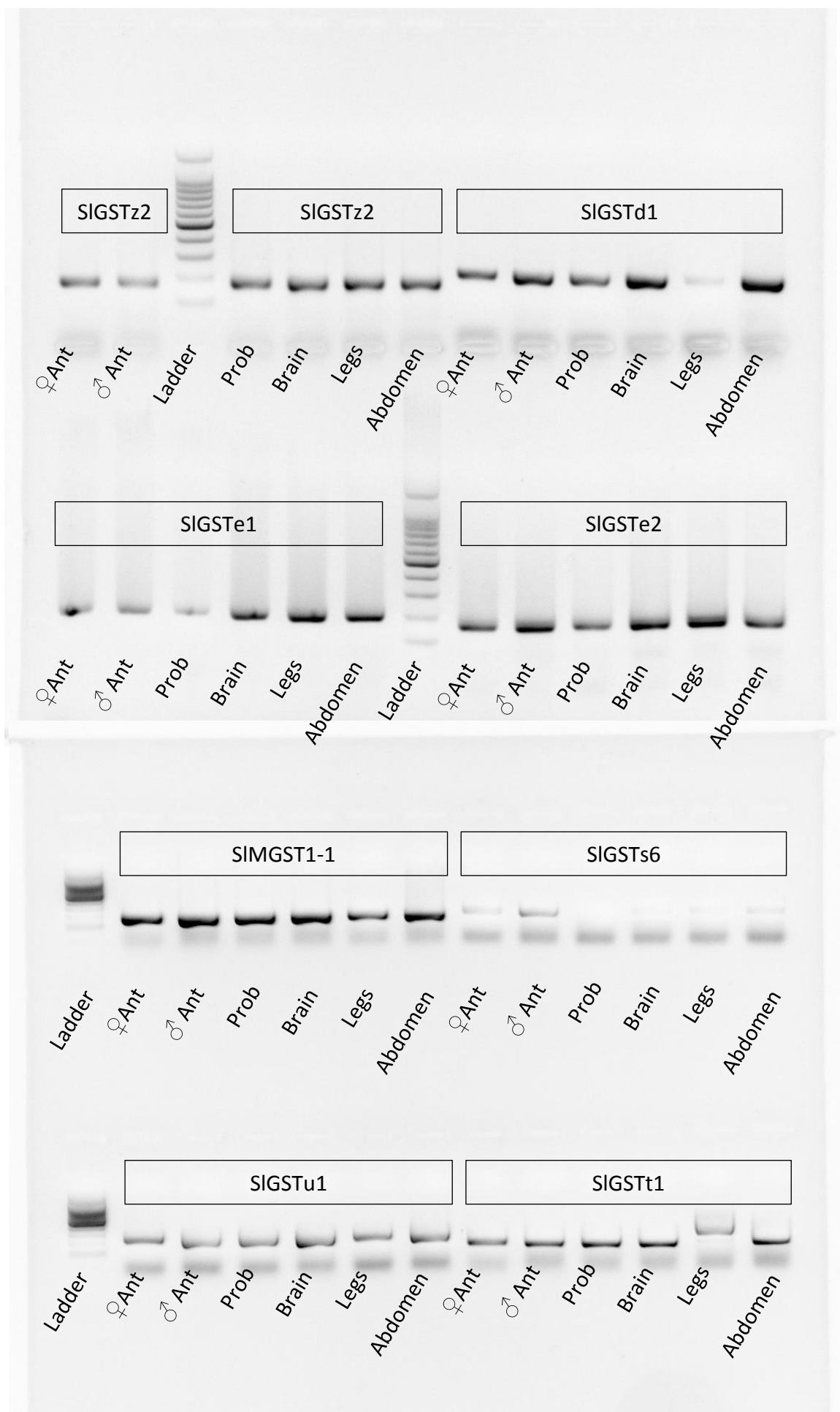

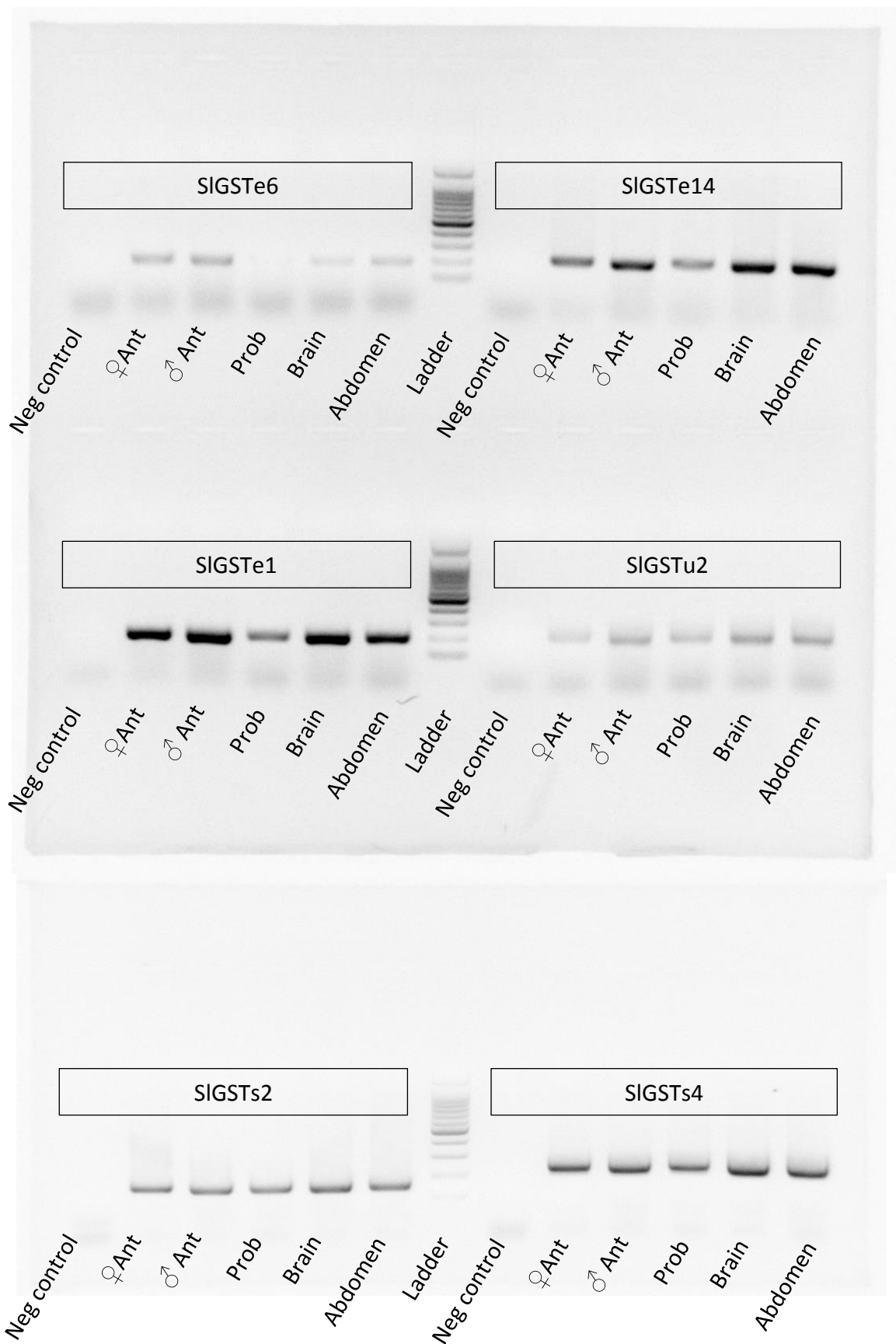

Larval PCR gels

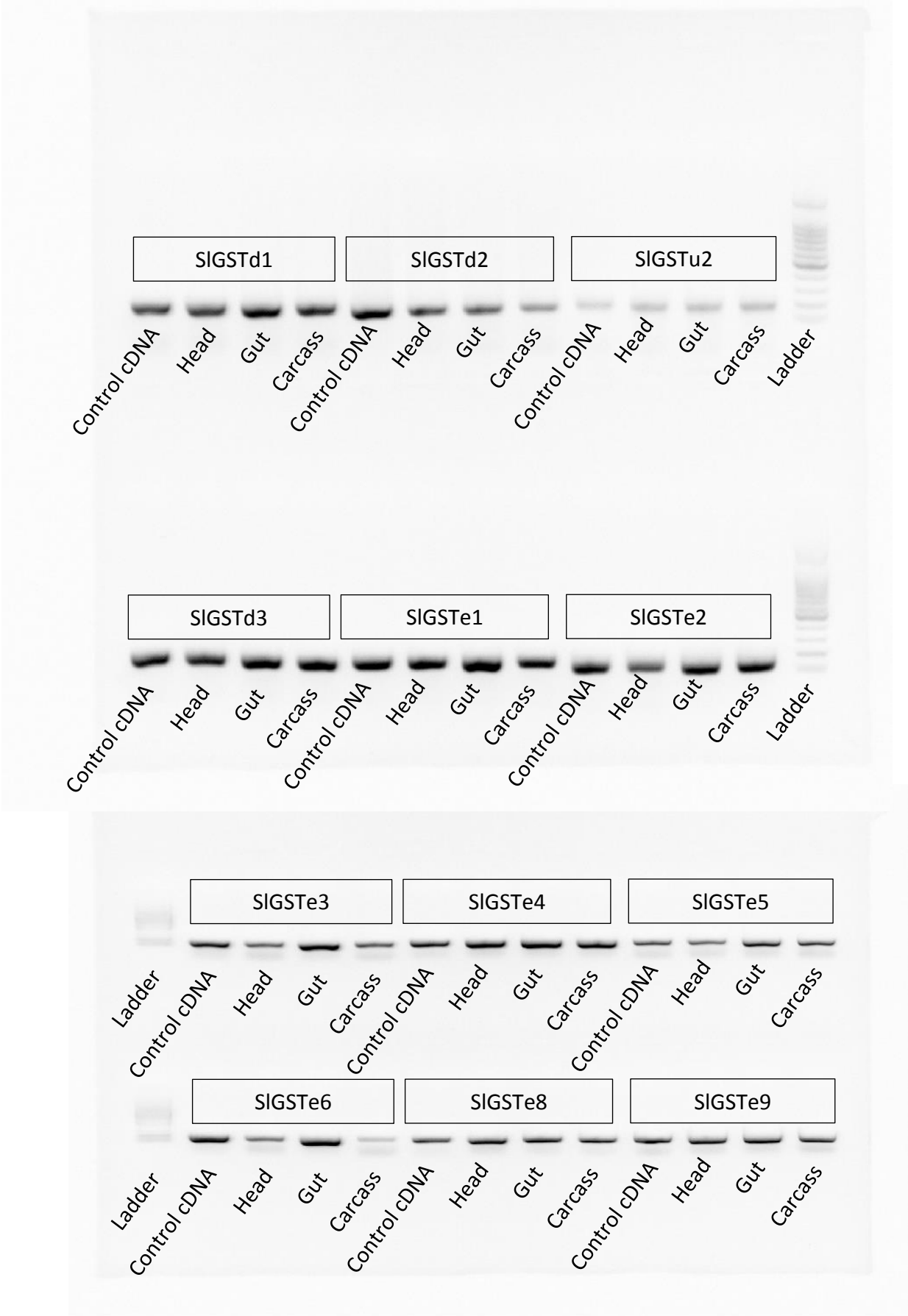

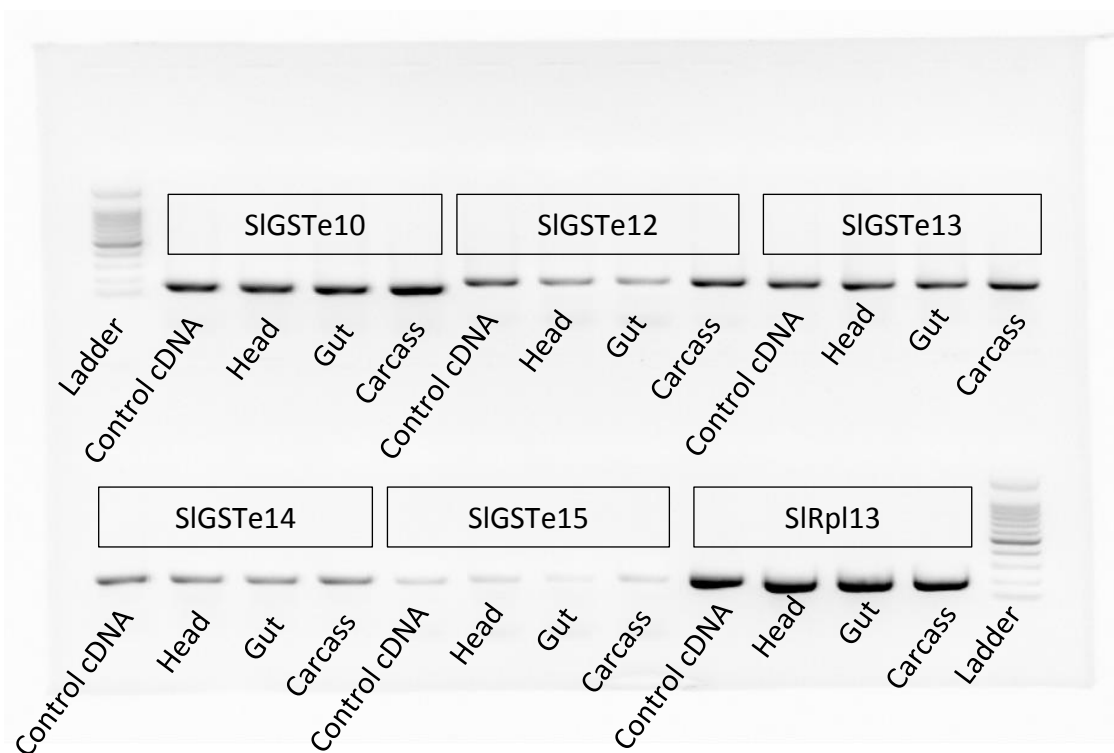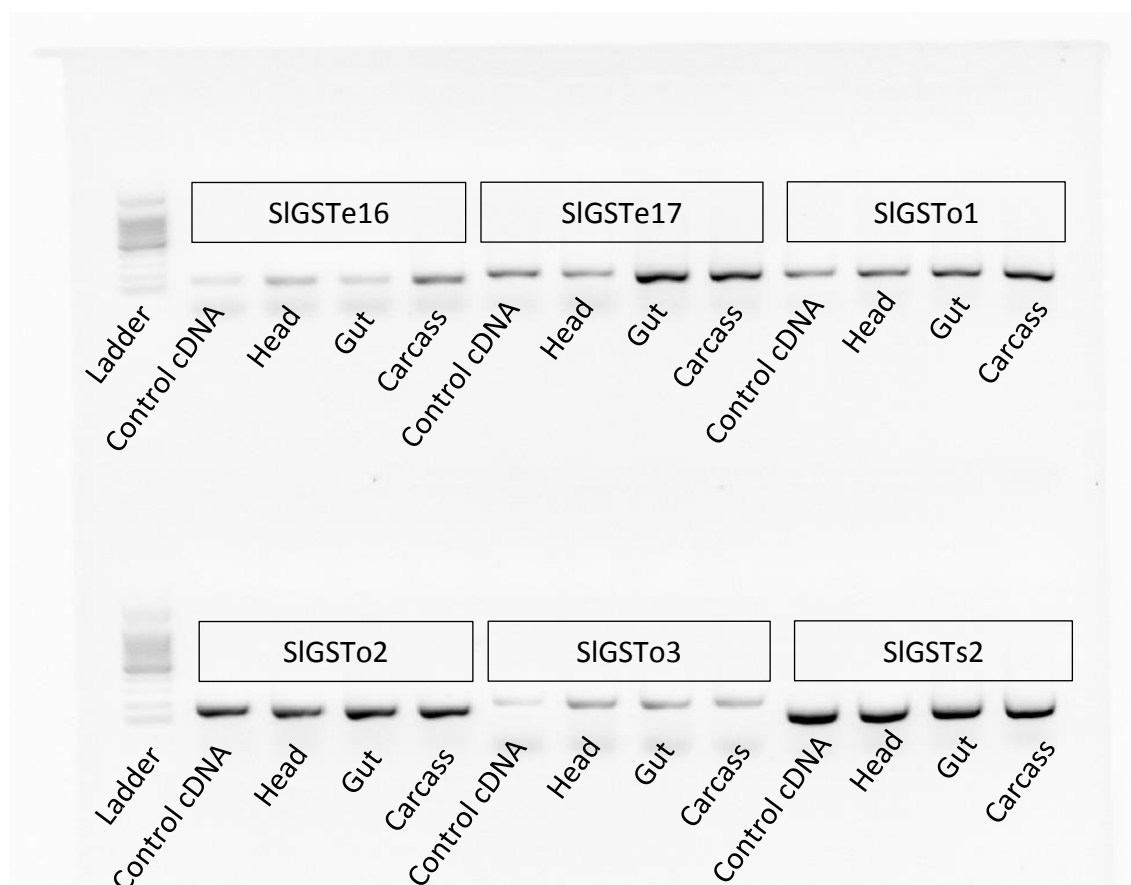

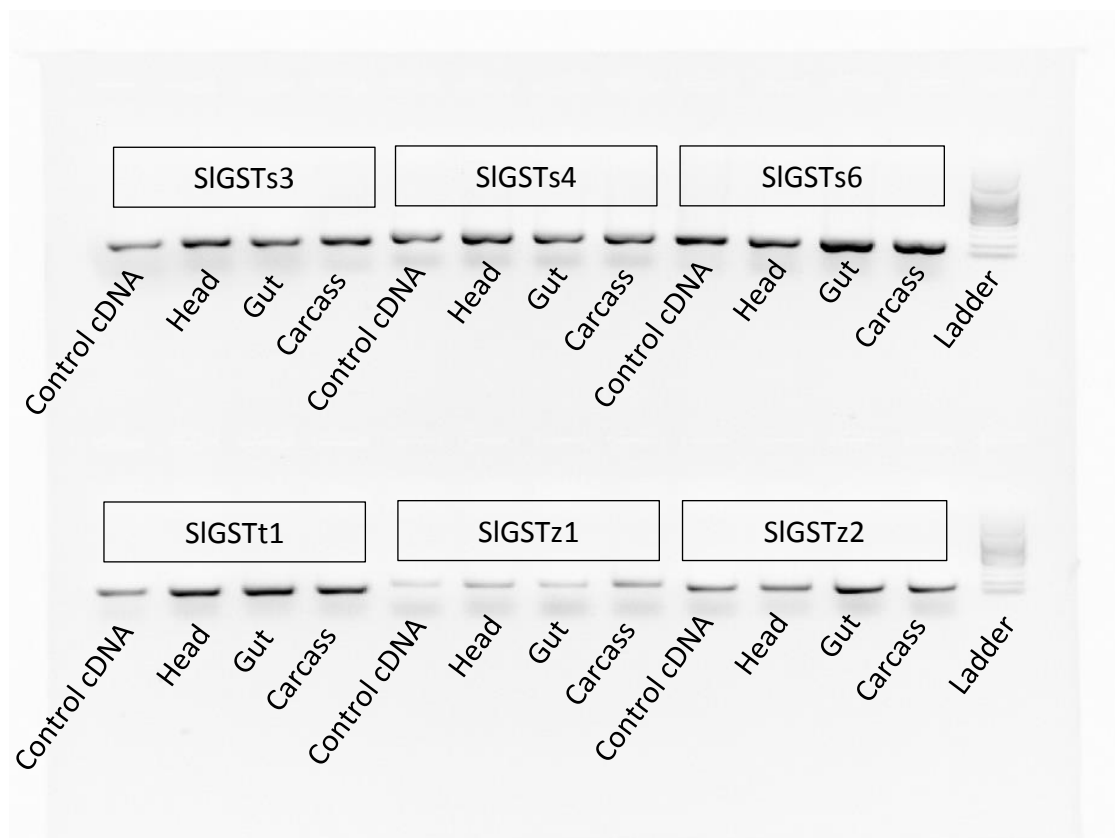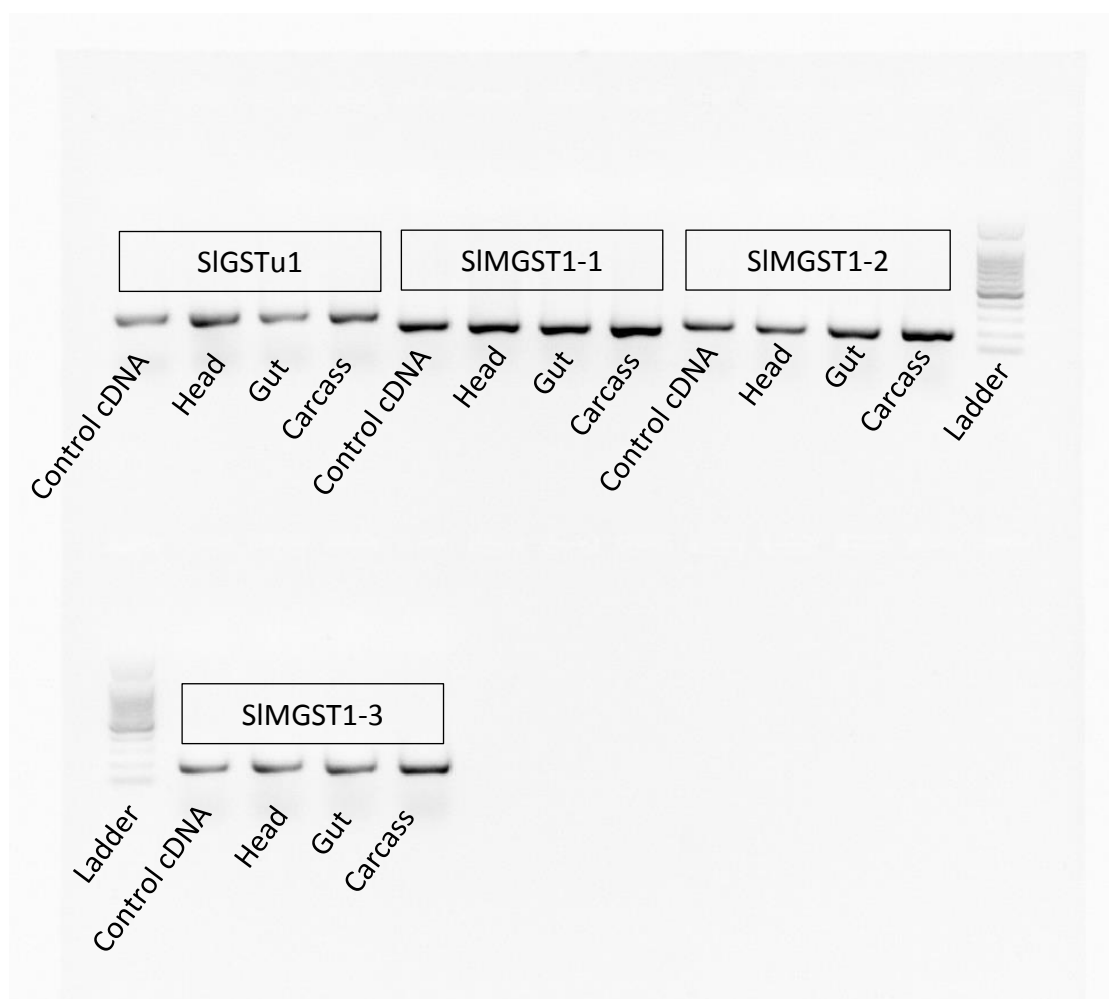

Supplement: Supplementary file 3 [file Data_Sheet_1.PDF]
